# Hypertension, antihypertensive drugs, and age at onset of Huntington’s disease

**DOI:** 10.1186/s13023-023-02734-1

**Published:** 2023-05-24

**Authors:** Yahui Zhu, Mao Li, Jiongming Bai, Haoran Wang, Xusheng Huang

**Affiliations:** 1 Medical School of Chinese PLA, Beijing, China; 2 Department of Neurology, the First Medical Center, Chinese PLA General Hospital, No. 28 Fuxing Road, Haidian District, Beijing, 100853 China; 3 College of Medicine, Nankai University, Tianjin, China

**Keywords:** Hypertension, Antihypertensive drugs, Huntington’s disease, Mendelian randomization

## Abstract

**Background:**

Associations between blood pressure (BP) with age at onset of Huntington’s disease (HD) have reported inconsistent findings. We used Mendelian randomization (MR) to assess effects of BP and lowering systolic BP (SBP) via the genes encoding targets of antihypertensive drugs on age at onset of HD.

**Methods:**

Genetic variants from genome-wide association studies(GWAS) of BP traits and BP-lowering variants in genes encoding antihypertensive drugs targets were extracted. Summary statistics for age at onset of HD were retrieved from the GWAS meta-analysis of HD residual age at onset from the GEM-HD Consortium included 9064 HD patients of European ancestry (4417 males and 4,647 females). MR estimates were calculated using the inverse variance weighted method, supplemented by MR-Egger, weighted median, and MR-PRESSO methods.

**Results:**

Genetically predicted SBP or diastolic BP increase was associated with a later age at onset of HD. However, after SBP/DBP was present as a covariate using multivariable MR method, no significant causal association was suggested. A 10-mm Hg reduction in SBP through variants in genes encoding targets of calcium channel blockers (CCB) was associated with an earlier age at onset of HD (β=-0.220 years, 95% CI =-0.337 to -0.102, P = 2.42 × 10^− 4^). We did not find a causal association between angiotensin converting enzyme inhibitors and β-blockers with the earlier HD onset. No heterogeneity and horizontal pleiotropy were identified.

**Conclusions:**

This MR analysis provided evidence that genetically determined SBP lowering through antihypertensive drugs might be associated with an earlier age at onset of HD. The results may have a potential impact on management of hypertension in the pre-motor-manifest HD population.

**Supplementary Information:**

The online version contains supplementary material available at 10.1186/s13023-023-02734-1.

## Background

Huntington’s disease (HD) is a neurodegenerative disease caused by the expansion of the cytosine-adenine-guanine (CAG) repeat in exon 1 of the huntingtin gene, resulting in the expression of mutant huntingtin (mHtt) containing extended polyglutamine [1–3]. The earliest and most pronounced brain change is striatal atrophy [4, 5]. HD is mainly characterized by cognitive, behavioral and motor deficits and the disease gradually worsens over time. The length of repeated expansion of CAG could account for approximately 50–70% variation of age at onset (AAO) of HD, and the remaining variations are explained by other genetic modifiers and environmental factors [6–9]. Considering that HD is a fatal disease with no effective treatment currently, it is important to identify interventions that can delay the age at onset of HD.

Hypertension is an important risk factor for cardiovascular events, and is one of the largest contributors to the global burden of diseases [10]. The persistence of hypertension and cardiovascular disease can negatively affect the structure and function of the brain [11–13]. There were relatively several studies on the effect of blood pressure on age at onset of HD, and the results were inconsistent. Valcárcel et al. [14] suggested that in the most common range of CAG expansion (the length of 40–44), HD patients with hypertension developed motor symptoms 5–8 years later than those without hypertension. Schultz et al. [15] argued that Valcarcel et al. did not account for the fact that the prevalence of hypertension increases with age. Using a large worldwide dataset (Enroll-HD) and controlling for confounding factors, Schultz et al. suggested that a diagnosis of hypertension may be associated with an earlier age of diagnosis of HD. However, Steventon et al. [16] used the same dataset (Enroll-HD) and controlled for confounders of age, sex, and BMI. They found that HD patients with hypertension had a later age at onset than normotensive patients. HD patients with hypertension were further divided into the untreated hypertension group and the treated group, and HD patients with treated hypertension had a later age of clinical onset compared with untreated hypertensive patients and normotensive individuals with HD. Steventon et al. suggested that these differences observed between normotensive and hypertensive HD patients appeared to be driven by the use of antihypertensive drugs. In particular, both Schultz et al. and Steventon et al. used the Enroll-HD, a very large longitudinal study of premanifest and manifest HD mutation carriers and controls. However, due to the different statistical methods used, the control for confounding factors and the definition of hypertension, different results were obtained.

In observational studies, Mendelian randomization(MR) is a novel method for assessing causal associations by using genetic variants [17]. Because genes are randomly assigned at conception, MR analysis overcomes the core deficiencies of observational studies and minimizes confounding bias and reverse causality. Therefore, MR can be used to assess potential causality [18]. Here, we performed Mendelian randomization to examine the effects of genetically determined blood pressure and genetic proxies for antihypertensive drug classes on age at onset of HD.

## Methods

### Standard protocol approvals and patient consents

This study followed the Strengthening the Reporting of Observational Studies in Epidemiology Using Mendelian Randomization (STROBE-MR) guide [19]. Since all analyses were performed using publicly available genome-wide association study (GWAS) summary data that had already obtained ethical review board approvals, no additional ethical permission was required from our institutional research ethics committees.

### Study design

**Fig. 1:**
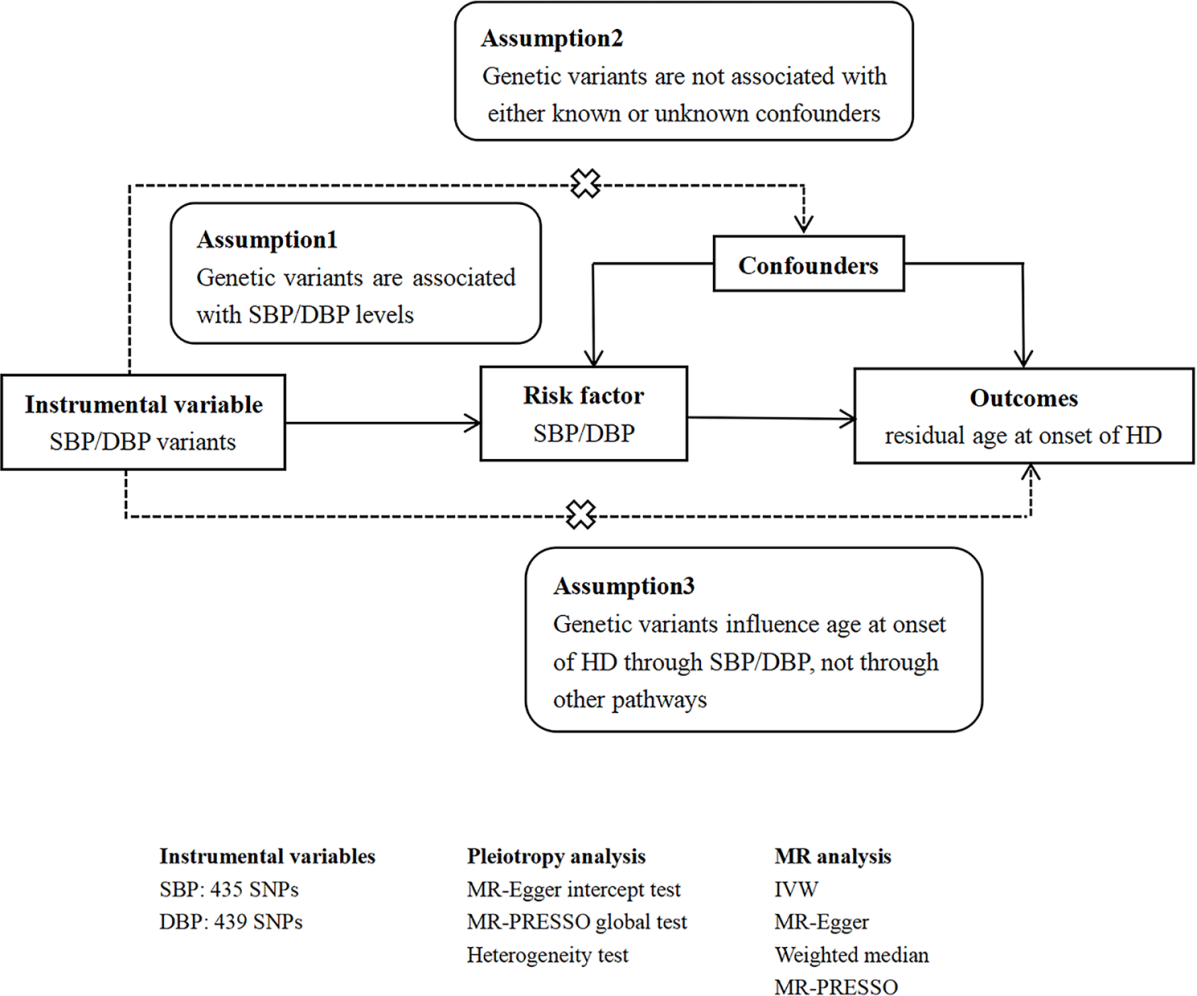
The flow chart of the MR study design SBP: systolic blood pressure; DBP: diastolic blood pressure; HD: Huntington’s disease; SNP: single nucleotide polymorphism; MR: Mendelian randomization; IVW: inverse variance weighted

In general, MR studies must satisfy three main assumptions: (1) the selected genetic variants are significantly associated with exposure; (2) the selected genetic variants are not associated with other confounders; (3) the selected genetic variants influence outcome only in the pathway of exposure. Both the second and third hypotheses are designed to ensure independence from pleiotropy. Here is the flow chart of our study design (Fig. 1).

### Genetic instrument selection

We used pooled data from a GWAS meta-analysis of blood pressure drawn from the International Consortium of Blood Pressure (ICBP) and the UK Biobank (UKB) on 757,601 Europeans [20]. ICBP GWAS data comprised 77 independent studies for up to 299,024 Europeans. The other 458,577 participants were from UKB. In the summary data, mean systolic blood pressure (SBP) and diastolic blood pressure (DBP) were 138.4 (SD 21.5) and 82.8 (SD 11.4) mm Hg, respectively. As genetic instruments for SBP and DBP, we selected single nucleotide polymorphisms (SNPs) associated with SBP or DBP at genome-wide significance level (p < 5 × 10^− 8^ ) and clumped using standard parameters (clumping window of 10000 kb, r^2^ < 0.001) to discard variants in linkage disequilibrium(LD). Subsequently, to satisfy the second hypothesis, we used the PhenoScanner tool [21] to screen whether the selected SNPs were associated with potential confounders affecting the age of HD onset. When using the PhenoScanner tool, the threshold for genome-wide significance was set at p < 5 × 10^− 8^. Finally, we assessed the power of SNPs using the F statistics (F = beta^2^/se^2^ ) for each SNP. To avoid weak instrumental variables, SNPs with less statistical power would be removed (F statistics < 10) [22]. The proportion of BP variance was explained by each instrument SNP R^2^, which was calculated using the following formula [23]: R^2^ = 2β^2^EAF(1–EAF) /SD^2^. Where EAF represents the effect allele frequency of the instrument SNP, and β denotes the effect size for SNP.

**Fig. 2:**
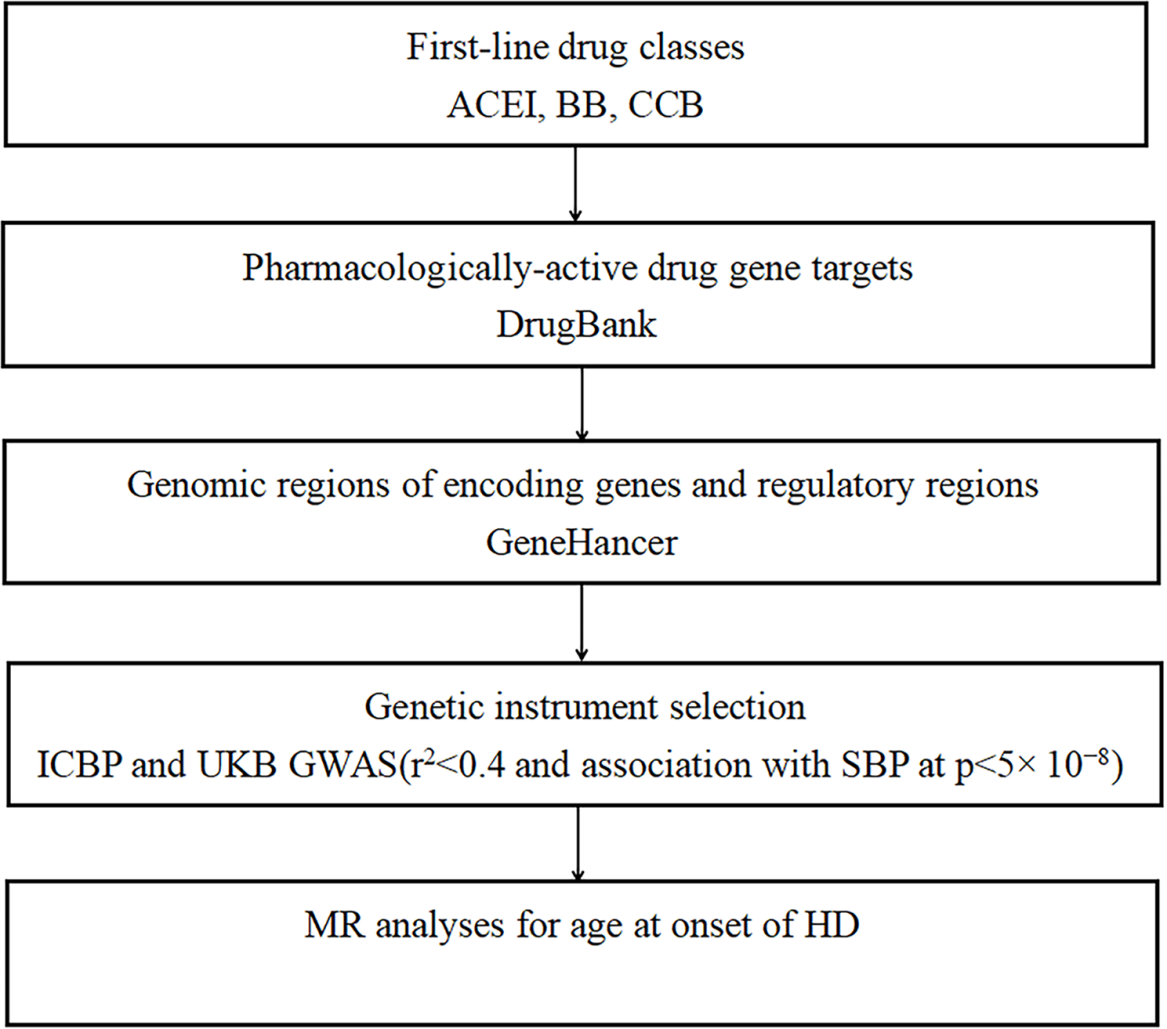
Selection strategy for genetic variants used as proxies for antihypertensive drug classes ACEI: angiotensin converting enzyme inhibitors; BB: beta blockers; CCB: calcium channel blockers; SBP: systolic blood pressure; HD: Huntington’s disease

We further selected genetic variants as proxies for SBP-lowering effects of common antihypertensive drugs (Fig. 2): angiotensin converting enzyme inhibitors (ACEI), β-blockers (BB) and calcium channel blockers (CCB). Based on the strategy previously described [24], we identified genes encoding pharmacologic targets associated with BP-lowering for common antihypertensive drug classes in DrugBank [25] and screened the corresponding genomic regions and regulatory regions (promoters and enhancers) of these genes [26]. Of all the variants identified from each gene, only variants that were significantly associated with SBP (P < 5 × 10^− 8^) and clumped to the LD threshold r^2^ < 0.4 in the 1000G European reference panel were considered as candidate proxies for each drug class. This relatively lenient LD correlation threshold can increase the proportion of variance explained, thus improving statistical power [27–29]. Then, we used more stringent LD threshold (r^2^ < 0.2) as an additional analysis of gene variation of antihypertensive drugs.

### Outcome data sources

The primary outcome in this study was residual age at onset of HD corrected for inherited CAG repeat length. The residual AAO of HD defined by the Genetic Modifiers of Huntington’s Disease (GeM-HD) Consortium. Age at onset of diagnostic motor signs and CAG repeat size based on the genotyping assay were used to calculate residual age at onset, representing years of deviation from the expectation [30]. For example, a HD subject with a residual age at onset of + 5 indicates an individual who developed motor symptoms 5 years later than expected considering their CAG repeat length. The GWAS meta-analysis of residual AAO from the GEM-HD Consortium included 9064 HD patients of European ancestry (4417 males and 4,647 females). In this study, their main aim was to find disease-modifying loci that act before clinical diagnosis, thereby delaying the onset of the disease.

### Statistical analysis

Causal effects were estimated with the random-effects inverse variance weighted (IVW) method. We applied five complementary methods (MR-Egger, weighted median, MR-PRESSO, simple mode, and weighted mode), which provided different assumptions about horizontal pleiotropy. The random-effects IVW method, the main method of the study, essentially assumed a zero intercept and performed a weighted regression of the SNP-exposure effects with the SNP-outcome effects. The MR Egger method provided more conservative estimates of causality in the presence of pleiotropy and was less likely to produce exaggerated test statistics [31]. Even if up to 50% of the information in the analysis came from invalid IV, the weighted median method could provide relatively valid estimates [32]. The MR-PRESSO method was used to detect outliers that might bias the results and to assess whether causal estimates change after removing outliers [33]. Due to the strong correlation between SBP and DBP, we also adopted the multivariable MR (MVMR) method to reduce the interference between them. Specifically, when assessing the causal relationship between SBP and AAO of HD, DBP was present as a covariate. However, SBP was included as a covariate when assessing the causal association between DBP and AAO of HD. The causal association was considered significant after correcting for multiple testing for two BP indexes [P < 0.025 (0.05/2)] and three antihypertensive drugs classes [P < 0.017 (0.05/3)].

Mendelian randomization associations between SBP/DBP and AAO of HD were scaled to 10 mm Hg increment in SBP and 5 mm Hg in DBP. Mendelian randomization associations between SBP-lowering effects of antihypertensive drug classes and AAO of HD were scaled to 10 mm Hg decrease in SBP.

Pleiotropy analyses were mainly based on three different statistical methods, including the MR-Egger intercept test [33], MR-PRESSO global test [33], and the heterogeneity test using Cochran’s Q statistic [34]. The above analyses showed statistically significant differences when P value < 0.05. Statistical analysis was performed in R version 4.1.2 (TwoSampleMR and MR-PRESSO packages).

## Results

### Genetically determined BP and age at onset of HD

**Fig. 3:**
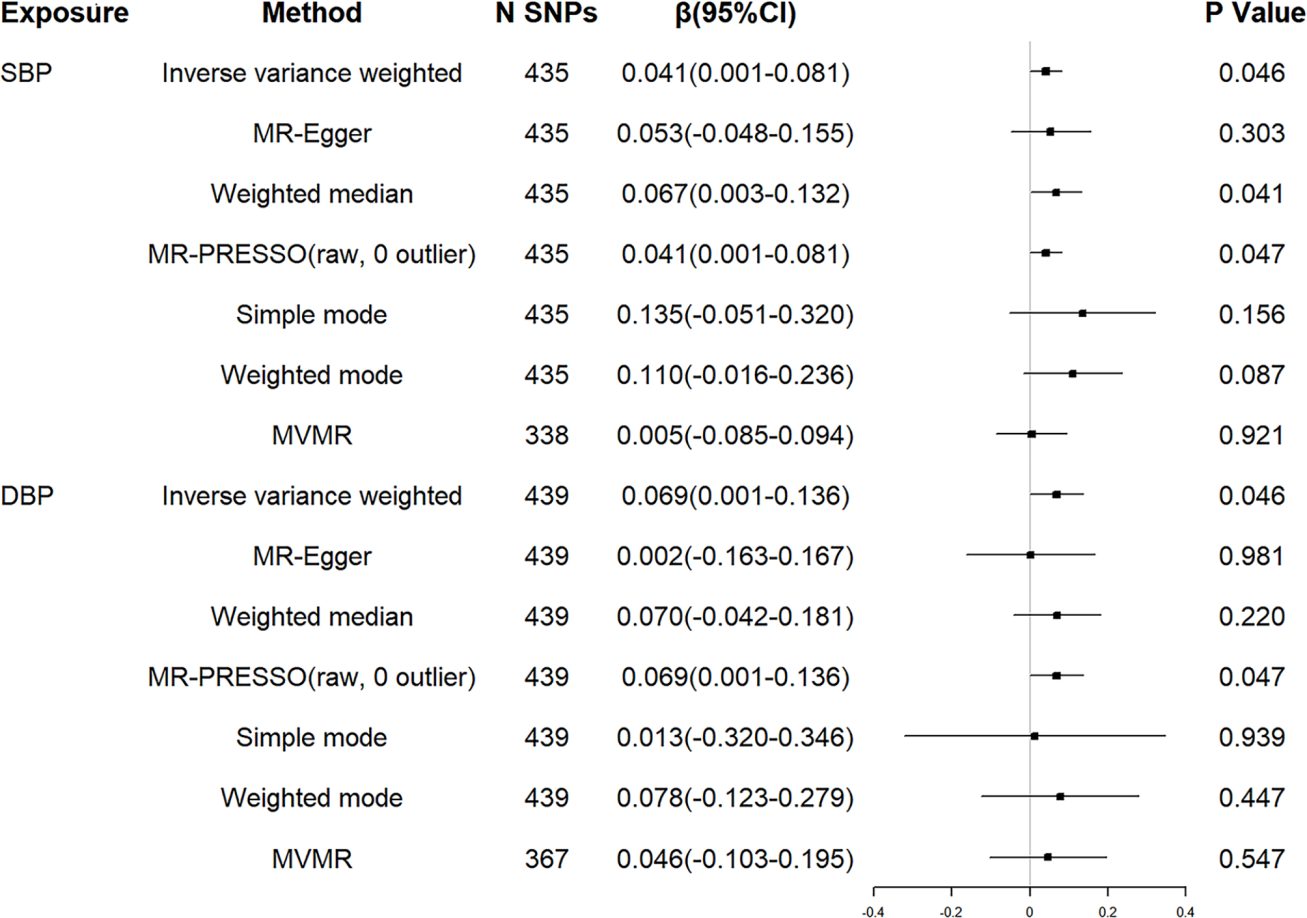
MR associations between genetically determined blood pressure and residual age at onset of HD. SBP: systolic blood pressure; DBP: diastolic blood pressure; HD: Huntington’s disease; SNP: single nucleotide polymorphism; CI: confidence interval

First, we examined the relationship between genetically determined BP and the age at onset of HD. No SNPs were associated with confounders, which were proved to have causal associations with AAO of HD, such as coffee consumption [35], lifetime smoking index [36], and telomere length [37]. The F statistics of each SNP were greater than the statistical threshold of 10, indicating that all SNPs had sufficient validity for subsequent analysis. Harmonising SBP/DBP and residual AAO of HD, 17/10 SNPs for being palindromic with intermediate allele frequencies were excluded. Thus, a total of 435/439 independent genetic variants associated with SBP/DBP were included in the downstream analyses, respectively (see Additional file 1 and 2). All these selected genetic variants could explain 1.59% /1.71% variance of SBP/DBP. Genetically predicted 10 mm Hg increment in SBP was associated with a later AAO of HD (β = 0.041 years, 95% confidence interval (CI) = 0.001 to 0.081, P = 0.046). Weighted median and MR-PRESSO analysis yielded similar pattern of effect estimates. In addition, 5 mm Hg increment in DBP was also associated with a later AAO of HD (β = 0.069 years, 95% CI = 0.001 to 0.136, P = 0.046) (Fig. 3). However, after SBP/DBP was present as a covariate using MVMR method, we observed no significant causal association between genetically determined BP and AAO of HD (Fig. 3).

Cochran Q statistic based on IVW (P = 0.464 for SBP; P = 0.490 for DBP) and MR-Egger (P = 0.452 for SBP; P = 0.487 for DBP) showed no evidence of heterogeneity. The MR Egger intercept (intercept = -0.004, P = 0.793 for SBP; intercept = 0.013, P = 0.386 for DBP) suggested no horizontal pleiotropy for instrumental variables, and the MR-PRESSO global test (P = 0.462 for SBP; P = 0.489 for DBP) supported that (see Table 1).

**Table 1:** Heterogeneity and pleiotropy tests of instrument effects

Exposure	N SNPs	Heterogeneity analysis				Pleiotropy analysis			
		Method	Q	Degree of freedom	P	Method	Egger intercept	SE	P
SBP	435	MR Egger	435.9	433	0.452	MR Egger intercept	-0.004	0.016	0.793
		IVW	436.0	434	0.464	MR-PRESSO Global test			0.462
DBP	439	MR Egger	437.3	437	0.487	MR Egger intercept	0.013	0.015	0.386
		IVW	438.1	438	0.490	MR-PRESSO Global test			0.489
BB (LD threshold, r^2^ < 0.4)	8	MR Egger	2.6	6	0.856	MR Egger intercept	-0.129	0.193	0.528
		IVW	3.1	7	0.879	MR-PRESSO Global test			0.889
CCB (LD threshold, r^2^ < 0.4)	58	MR Egger	52.9	56	0.594	MR Egger intercept	0.065	0.051	0.207
		IVW	54.5	57	0.569	MR-PRESSO Global test			0.616
BB(LD threshold, r^2^ < 0.2)	7	MR Egger	2.0	5	0.852	MR Egger intercept	-0.115	0.194	0.580
		IVW	2.3	6	0.887	MR-PRESSO Global test			0.884
CCB(LD threshold, r^2^ < 0.2)	30	MR Egger	33.0	28	0.236	MR Egger intercept	0.017	0.072	0.813
		IVW	33.1	29	0.276	MR-PRESSO Global test			0.303

**SNP: single nucleotide polymorphism; MR: Mendelian randomization; IVW: inverse variance weighted; SBP: systolic blood pressure; DBP: diastolic blood pressure; CCB: calcium channel blocker; BB: β-blockers; SE: standard error**

### Genetic proxies for antihypertensive drugs and AAO of HD

Next, we selected BP-lowering variants in genes encoding drug targets as proxies for the effects of antihypertensive drug classes and examined their effects on AAO of HD. We identified 1 proxy for ACEI, 8 for BB, and 60 for CCB (see Additional file 3). No SNPs were found to be associated with possible confounders using the PhenoScanner tool. The F values of all SNPs were greater than 10, proving that it was unlikely to produce weak instrumental variables bias.

**Fig. 4:**
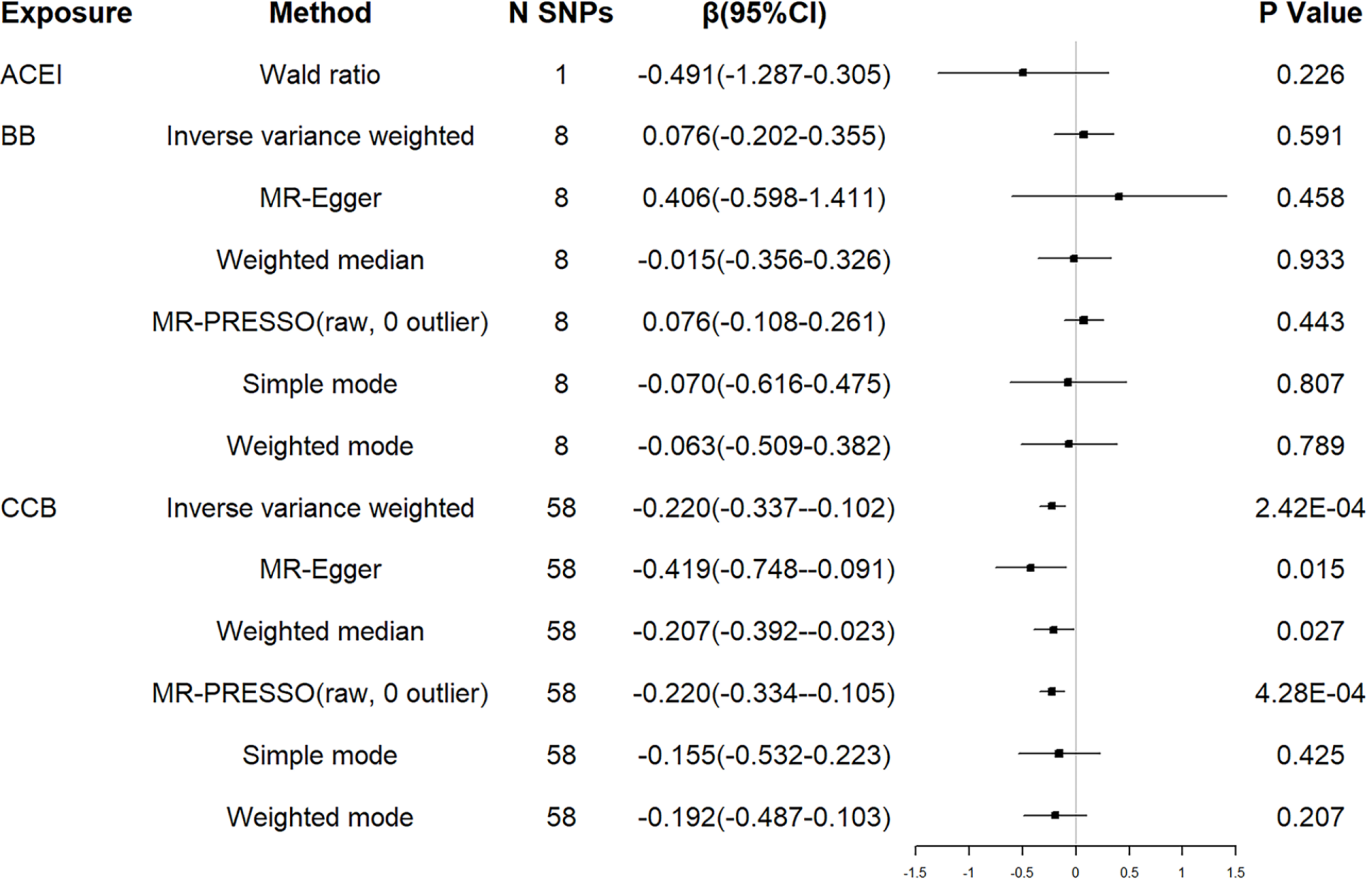
MR associations between genetic proxies for antihypertensive drug classes and residual age at onset of HD (LD r^2^ < 0.4) ACEI: angiotensin converting enzyme inhibitors; BB: beta blockers; CCB: calcium channel blockers; HD: Huntington’s disease; SNP: single nucleotide polymorphism; CI: confidence interval; LD: linkage disequilibrium

A 10-mm Hg reduction in SBP through variants in genes encoding targets of CCB was associated with an earlier AAO of HD (β=-0.220 years, 95% CI =-0.337 to -0.102, P = 2.42 × 10^− 4^). The associations were confirmed using the methods of weighted median and MR Egger. However, no causal relationship between ACEI/BB and AAO of HD was suggested (see Fig. 4).

The Cochran Q statistic of IVW method ( P = 0.569 for CCB; P = 0.879 for BB) and MR Egger ( P = 0.594 for CCB; P = 0.856 for BB) indicated no notable heterogeneity across instrument SNP effects. The MR Egger intercept analysis did not show evidence of horizontal pleiotropy (intercept = 0.065, P = 0.207 for CCB; intercept = -0.129, P = 0.528 for BB), and the MR-PRESSO global test (P = 0.616 for CCB; P = 0.889 for BB) supported that (see Table 1).

### Results of additional analysis

**Fig. 5:**
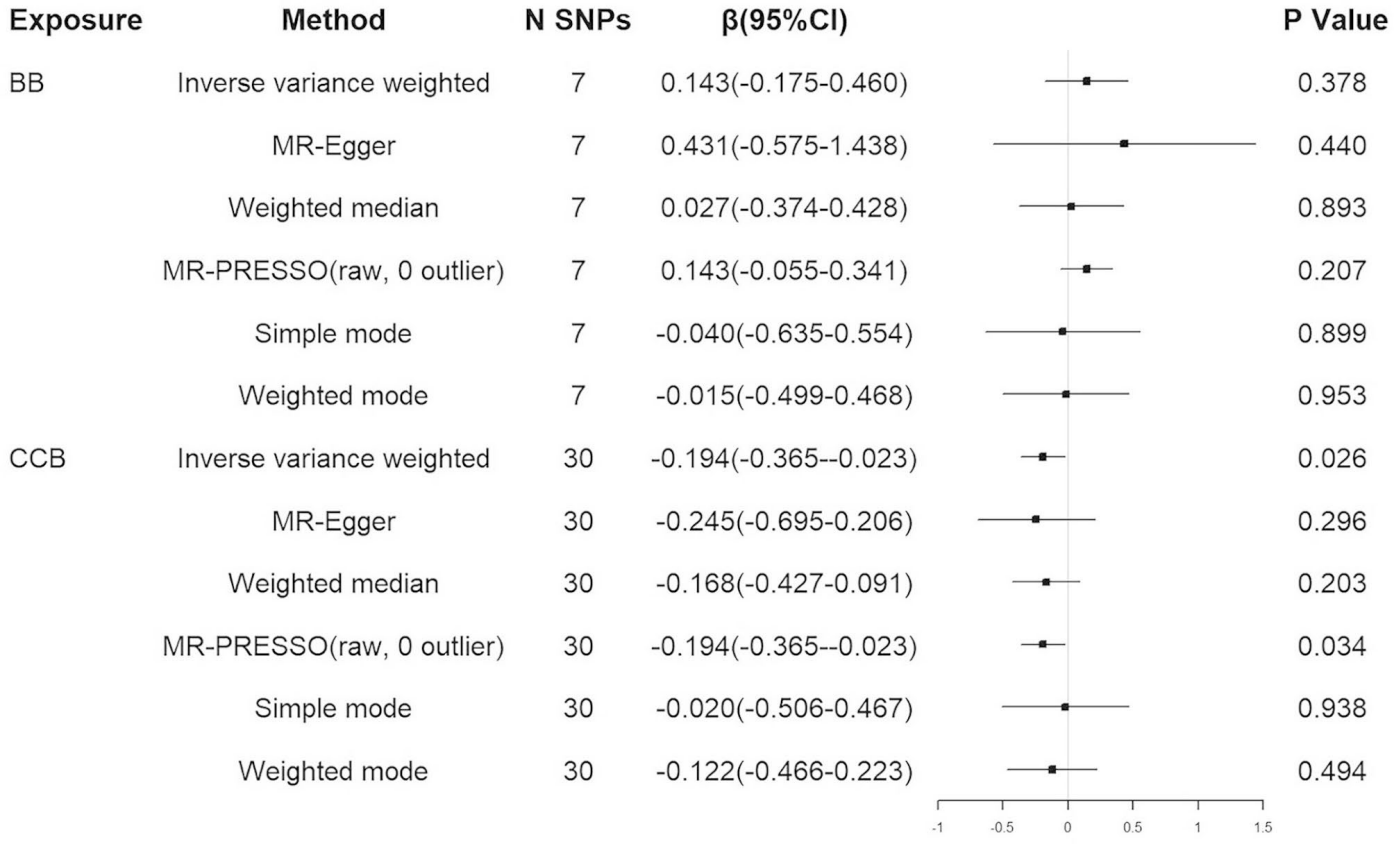
MR associations between genetic proxies for antihypertensive drug classes and residual age at onset of HD (LD r^2^ < 0.2) BB: beta blockers; CCB: calcium channel blockers; HD: Huntington’s disease; SNP: single nucleotide polymorphism; CI: confidence interval; LD: linkage disequilibrium

Additional analysis restricted to the set of SNPs with the LD threshold (r^2^ < 0.2) showed consistent association estimates (β=-0.194 years, 95% CI = -0.365 to -0.023, P = 0.026; Fig. 5) for CCB. The results of MR-PRESSO analysis showed similar tendency. Similarly, there was no evidence to support a causal association between BB and AAO of HD. No notable heterogeneity and horizontal pleiotropy of CCB and BB were detected (see Table 1).

## Discussion

In this MR study, we explored the causal relationship between blood pressure, antihypertensive drugs and AAO of HD. We used genetic variants associated with BP as proxies to provide suggestive evidence that genetic exposure to SBP lowering through antihypertensive drugs might be associated with an earlier age of HD onset.

Currently, there were some studies on the relationship between hypertension and AAO of HD, and the findings were mixed [14–16]. Previous observational studies have shown that hypertension might lead to an earlier or later onset of HD. More interestingly, the two studies [15, 16] used the same large worldwide dataset (Enroll-HD), however, due to differences in HD patients enrolled, statistical methods, control for confounding factors, and definitions of hypertension, leading to completely opposite conclusions. Since Enroll-HD was an observational study and lacked BP measurements and degree of BP control, it was difficult to assess the causal relationship between actual blood pressure and AAO of HD. Large-scale biobank datasets can provide an unparalleled opportunity for assumption-free causal inference. Through MR analysis, we found no significant causal association between genetically predicted SBP/DBP and AAO of HD.

Observational studies suggested that treatment with antihypertensive drugs might delay the onset of HD [15, 16]. Unfortunately, these observational studies did not include vital sign data. Most participants were treated with antihypertensive drugs, but the effects of treatment and extent of BP control were unknown. The association between treatment with antihypertensive agents and delayed HD onset could not be accurately assessed. Moreover, there were no relevant high-quality interventional studies to evaluate the effects of antihypertensive drug treatment on the the age at onset of HD. Using MR, the current study expanded previous evidence using genetic variants in a very large cohort of well-characterized study participants. The study showed that genetically determined BP lowering through antihypertensive drugs was associated with an earlier AAO of HD, and CCB might not be considered a promising preventive strategy to delay the onset of HD.

Glodzik et al. [38] found that a decline in blood pressure was related to dementia and brain damage, and that these associations were primarily found in individuals treated with antihypertensive drugs. In longstanding hypertension, the limits of cerebral blood flow regulation are altered and the mean arterial pressure threshold for maintaining cerebral blood flow is shifted to higher levels [39]. This change may increase the brain’s vulnerability to hypoperfusion at lower BP values [40], indicating that higher pressure is needed to maintain adequate blood flow. Animal studies have shown that even mild and transient hypoperfusion results in increased tau phosphorylation and long lasting increases in amyloid beta [41]. In this study, we thought that lowering blood pressure in hypertensive patients with impairment of cerebral autoregulation might be detrimental to brain perfusion and oxygenation, thus, further promoting the accumulation of mutant huntingtin in neurons and contributing to the development of HD. Our results do not suggest that pre-motor-manifest HD patients need to increase blood pressure to delay the onset, but rather that pre-motor-manifest HD patients with hypertension should be cautious in antihypertensive treatment.

As for the effect of antihypertensive drugs on the age at onset of HD, the study could only assess the effect of lowering blood pressure rather than other mechanisms on HD. However, we need to pay attention to the neuroprotective effects of antihypertensive drugs, including calcium channel blockers, probably not by treating hypertension per se. For example, calcium accumulation in neurons might be neurotoxic and thus CCB might have a neuroprotective effect [42]. Moreover, a recent study did report that the use of felodipine in mice might induce autophagy and had benefits for neurodegenerative diseases, including HD, and these benefits were independent of the cardiovascular system [43]. In the future, a prospective cohort of large-scale population-based interventions could be established to assess the effects of different antihypertensive agents on age at HD onset, in order to distinguish whether it is through the effects of lowering blood pressure or other mechanisms, such as neuroprotective effects. Combining these data with the results generated by the MR framework may provide compelling conclusions. Although the mechanisms underlying the effects of antihypertensive drugs on earlier HD onset remain unclear and warrant further investigation, MR analysis certainly offers great promise for identifying risk or protective factors in the era of large-scale genetic epidemiology.

The advantages of our study included the following aspects. First, we provided evidence that hypertension might delay the onset of HD, and the use of antihypertensive medications might be associated with an earlier age at onset of HD using genetic data for the first time. Second, we used a variety of methods to verify the accuracy of the results, such as calculating the F statistic to exclude weak instrumental variable bias and performing heterogeneity and pleiotropy tests to ensure the reliability of the MR results.

However, the results of this study should be interpreted in conjunction with some limitations. First, our study was limited by the fact that MR studies the effects of lifetime exposure, whereas drugs are typically exposed for much shorter periods, and blood pressure may have age-dependent effects. The effect sizes we estimated were not representative of the relationship between critical periods of exposures and outcomes. It can also be particularly problematic if a drug’s protein target is beneficial at one point in the life course and detrimental at another. Therefore, further research into the effect of BP and antihypertensive drugs on the AAO of HD is recommended, especially randomized controlled trials, and to explore how the effects vary with age. Second, because the drug target model only focuses on the target effect of the specific treatment, our drug target genetic results do not reflect the pharmacokinetics of drug use. Third, we did not explore the causal relationship of other antihypertensive drugs, including ACEI and BB. These null results did not imply that these drugs were not detrimental, possibly given that the limited number of SNPs included did not provide sufficient statistical power for meaningful analysis. Future studies including larger BP GWAS datasets might provide deeper insights into the effect of different types of BP-lowering drugs on the AAO of HD. Fourth, the estimated effect of blood pressure on AAO of HD, which is associated with higher mortality, may be susceptible to survival bias. Finally, since all participants were of European ancestry, it might be difficult to extrapolate the results to other ethnic groups.

## Conclusions

In this two-sample MR study, we examined the effect of blood pressure and lowering blood pressure with antihypertensive drugs on AAO of HD using genetic variants. We provided evidence that genetically determined BP lowering through antihypertensive drugs might be associated with an earlier age at onset of HD. Moreover, CCB may not be a promising strategy for delaying HD onset. Our results complemented the findings of observational studies and might have a potential impact on management of hypertension in the pre-motor-manifest HD population. However, further clinical randomized controlled trials are needed. In addition, for pre-HD patients with hypertension, the probability of cardiovascular and cerebrovascular diseases should be taken into account, and the use of antihypertensive drugs should be evaluated comprehensively.

## Electronic supplementary material

Below is the link to the electronic supplementary material.

Supplementary Material 1

Supplementary Material 2

Supplementary Material 3

## Data Availability

This study was based on summary statistics. The GWAS data from the ICBP and UKB meta-analysis are publicly available(https://gwas.mrcieu.ac.uk/). The age at onset of HD GWAS data are available from the corresponding authors upon request.
