# Supplementary material for: Hypertension, antihypertensive drugs, and age at onset of Huntington’s disease
Source: Orphanet J Rare Dis. 2023 May 24;18:125. doi: 10.1186/s13023-023-02734-1 (PMC10207760; doi:10.1186/s13023-023-02734-1)
Supplement: Supplementary file 1 — Supplementary Material 1 [file 13023_2023_2734_MOESM1_ESM.docx]

**Table S1 The characteristics of the selected SBP instrumental variables**

| **SNP** | **Chr** | **Position**  **(GRCh37/hg19)** | **Effect allele** | **Other allele** | **EAF** | **beta** | **SE** | **P-value** | **R^2^** | **F** |
| --- | --- | --- | --- | --- | --- | --- | --- | --- | --- | --- |
| rs1000423 | 17 | 59475642 | T | C | 0.7316 | 0.4138 | 0.0346 | 6.50E-33 | 7.41E-05 | 143.0 |
| rs10045307 | 5 | 127411454 | G | C | 0.227 | 0.2024 | 0.0362 | 2.21E-08 | 1.45E-05 | 31.3 |
| rs10048404 | 18 | 54578482 | T | C | 0.3701 | -0.2607 | 0.0317 | 1.91E-16 | 4.16E-05 | 67.6 |
| rs10048760 | 2 | 174977976 | G | T | 0.4712 | 0.1862 | 0.0301 | 6.56E-10 | 2.52E-05 | 38.3 |
| rs1006545 | 10 | 102553647 | T | G | 0.8872 | 0.6846 | 0.048 | 3.50E-46 | 5.37E-05 | 203.4 |
| rs10069690 | 5 | 1279790 | T | C | 0.2582 | 0.3098 | 0.0369 | 4.47E-17 | 3.56E-05 | 70.5 |
| rs1010064 | 12 | 20000315 | C | A | 0.1837 | -0.3571 | 0.0387 | 3.02E-20 | 3.37E-05 | 85.1 |
| rs10188003 | 2 | 66773469 | T | C | 0.393 | 0.1883 | 0.0307 | 8.80E-10 | 2.37E-05 | 37.6 |
| rs10207726 | 2 | 112744260 | T | C | 0.296 | -0.2142 | 0.033 | 8.06E-11 | 2.32E-05 | 42.1 |
| rs10224210 | 7 | 151413194 | C | T | 0.2789 | 0.3831 | 0.034 | 1.60E-29 | 6.74E-05 | 127.0 |
| rs10282122 | 7 | 2529623 | T | C | 0.6684 | -0.302 | 0.0327 | 2.46E-20 | 4.99E-05 | 85.3 |
| rs10420519 | 19 | 45298461 | T | G | 0.0347 | -0.4921 | 0.0887 | 2.86E-08 | 2.72E-06 | 30.8 |
| rs1044822 | 2 | 230629138 | T | C | 0.1482 | -0.248 | 0.0424 | 5.16E-09 | 1.14E-05 | 34.2 |
| rs10460108 | 18 | 73034151 | G | A | 0.5199 | -0.2141 | 0.0301 | 1.12E-12 | 3.33E-05 | 50.6 |
| rs1049212 | 16 | 4932929 | G | A | 0.5693 | 0.299 | 0.0302 | 4.59E-23 | 6.35E-05 | 98.0 |
| rs10501122 | 11 | 30192151 | C | T | 0.361 | -0.1916 | 0.0315 | 1.18E-09 | 2.25E-05 | 37.0 |
| rs10501410 | 11 | 72088806 | A | G | 0.0692 | 0.4122 | 0.0607 | 1.10E-11 | 7.84E-06 | 46.1 |
| rs1052501 | 3 | 41925398 | T | C | 0.8329 | 0.2262 | 0.0412 | 4.14E-08 | 1.11E-05 | 30.1 |
| rs10746963 | 9 | 77238558 | G | A | 0.8166 | 0.2177 | 0.0388 | 2.05E-08 | 1.24E-05 | 31.5 |
| rs10749572 | 10 | 82136664 | T | G | 0.5444 | -0.203 | 0.0302 | 1.88E-11 | 2.96E-05 | 45.2 |
| rs10750441 | 11 | 130469044 | T | C | 0.6621 | 0.1754 | 0.0319 | 3.74E-08 | 1.79E-05 | 30.2 |
| rs10776752 | 1 | 113044328 | T | G | 0.0809 | 0.8211 | 0.0576 | 4.61E-46 | 3.99E-05 | 203.2 |
| rs10777213 | 12 | 90349999 | A | G | 0.5244 | -0.1786 | 0.0299 | 2.45E-09 | 2.35E-05 | 35.7 |
| rs10782230 | 6 | 126228512 | A | G | 0.4845 | 0.2106 | 0.0302 | 2.91E-12 | 3.21E-05 | 48.6 |
| rs10804330 | 2 | 227185749 | C | T | 0.4332 | -0.2351 | 0.0306 | 1.62E-14 | 3.83E-05 | 59.0 |
| rs10866828 | 8 | 23401534 | T | C | 0.2496 | 0.2476 | 0.0355 | 3.19E-12 | 2.41E-05 | 48.6 |
| rs10914124 | 1 | 180865798 | C | T | 0.3833 | -0.234 | 0.0312 | 6.32E-14 | 3.51E-05 | 56.3 |
| rs10941043 | 5 | 33194751 | G | T | 0.2902 | 0.2585 | 0.0332 | 6.42E-15 | 3.30E-05 | 60.6 |
| rs10980408 | 9 | 113249071 | C | T | 0.0359 | 0.7606 | 0.0827 | 3.83E-20 | 7.73E-06 | 84.6 |
| rs11097909 | 4 | 106911321 | C | T | 0.8528 | 0.3628 | 0.043 | 3.35E-17 | 2.36E-05 | 71.2 |
| rs11120093 | 1 | 207211326 | T | C | 0.4082 | -0.1792 | 0.0307 | 5.13E-09 | 2.17E-05 | 34.1 |
| rs11145807 | 9 | 139520789 | G | A | 0.5943 | -0.2135 | 0.0322 | 3.54E-11 | 2.80E-05 | 44.0 |
| rs11159091 | 14 | 75074316 | A | G | 0.4615 | 0.1978 | 0.0303 | 6.79E-11 | 2.80E-05 | 42.6 |
| rs111866816 | 10 | 94441507 | T | C | 0.0709 | 0.3569 | 0.0597 | 2.29E-09 | 6.22E-06 | 35.7 |
| rs11191580 | 10 | 104906211 | C | T | 0.0824 | -1.0995 | 0.055 | 7.74E-89 | 7.98E-05 | 399.6 |
| rs11210029 | 1 | 41865293 | G | A | 0.3678 | 0.203 | 0.0313 | 8.92E-11 | 2.58E-05 | 42.1 |
| rs11222084 | 11 | 130273230 | T | A | 0.3621 | 0.3363 | 0.0316 | 1.80E-26 | 6.91E-05 | 113.3 |
| rs11241313 | 5 | 114428167 | T | C | 0.3112 | -0.2071 | 0.0326 | 2.23E-10 | 2.28E-05 | 40.4 |
| rs112509803 | 7 | 24735004 | C | G | 0.1138 | -0.2641 | 0.0477 | 3.18E-08 | 8.16E-06 | 30.7 |
| rs11252324 | 10 | 4124568 | T | G | 0.0771 | -0.4164 | 0.0573 | 3.61E-13 | 9.92E-06 | 52.8 |
| rs113086489 | 17 | 7171356 | T | C | 0.5525 | 0.3249 | 0.0307 | 3.80E-26 | 7.31E-05 | 112.0 |
| rs113264678 | 22 | 30135079 | T | C | 0.046 | 0.4063 | 0.0727 | 2.26E-08 | 3.62E-06 | 31.2 |
| rs1133400 | 10 | 134459388 | G | A | 0.214 | 0.2975 | 0.0376 | 2.53E-15 | 2.78E-05 | 62.6 |
| rs115262049 | 2 | 43196694 | T | A | 0.0868 | -0.5893 | 0.0552 | 1.29E-26 | 2.38E-05 | 114.0 |
| rs1154214 | 18 | 24546824 | G | T | 0.6037 | 0.2031 | 0.0306 | 3.27E-11 | 2.78E-05 | 44.1 |
| rs11592107 | 10 | 122968964 | A | G | 0.3096 | 0.3024 | 0.0326 | 1.55E-20 | 4.86E-05 | 86.0 |
| rs11604357 | 11 | 45351729 | A | C | 0.1622 | -0.277 | 0.0411 | 1.60E-11 | 1.63E-05 | 45.4 |
| rs11636952 | 15 | 75114322 | C | T | 0.6859 | -0.5313 | 0.0328 | 4.22E-59 | 0.000149228 | 262.4 |
| rs11641374 | 16 | 1347717 | A | C | 0.5995 | -0.1943 | 0.0309 | 3.26E-10 | 2.51E-05 | 39.5 |
| rs11653927 | 17 | 2012094 | T | C | 0.3845 | -0.2796 | 0.0308 | 1.17E-19 | 5.15E-05 | 82.4 |
| rs11655604 | 17 | 79365861 | T | C | 0.3579 | -0.2033 | 0.0333 | 1.09E-09 | 2.26E-05 | 37.3 |
| rs11672660 | 19 | 46180184 | T | C | 0.1996 | 0.2212 | 0.0381 | 6.32E-09 | 1.42E-05 | 33.7 |
| rs1169078 | 12 | 122416254 | G | C | 0.3121 | 0.1971 | 0.0327 | 1.68E-09 | 2.06E-05 | 36.3 |
| rs117206641 | 12 | 133086888 | T | C | 0.1108 | 0.3154 | 0.0499 | 2.66E-10 | 1.04E-05 | 40.0 |
| rs117285318 | 17 | 7870642 | C | T | 0.0775 | -0.4413 | 0.0589 | 6.93E-14 | 1.06E-05 | 56.1 |
| rs117464403 | 10 | 107158054 | A | G | 0.0183 | 0.864 | 0.1199 | 5.80E-13 | 2.46E-06 | 51.9 |
| rs11847049 | 14 | 69259406 | G | C | 0.2166 | 0.2272 | 0.0364 | 4.44E-10 | 1.75E-05 | 39.0 |
| rs11874246 | 18 | 42596789 | T | C | 0.2963 | 0.2856 | 0.0328 | 3.22E-18 | 4.17E-05 | 75.8 |
| rs11925504 | 3 | 14943965 | A | G | 0.5721 | -0.2901 | 0.0305 | 1.78E-21 | 5.85E-05 | 90.5 |
| rs11960210 | 5 | 157817634 | C | T | 0.3755 | -0.4727 | 0.0313 | 1.25E-51 | 0.000141193 | 228.1 |
| rs11977526 | 7 | 46008110 | A | G | 0.4009 | -0.3213 | 0.0312 | 6.62E-25 | 6.72E-05 | 106.1 |
| rs1199330 | 3 | 138101529 | G | A | 0.1176 | 0.2654 | 0.047 | 1.65E-08 | 8.74E-06 | 31.9 |
| rs12042924 | 1 | 197297417 | C | T | 0.4716 | 0.1807 | 0.0303 | 2.62E-09 | 2.34E-05 | 35.6 |
| rs12063372 | 1 | 59621911 | A | G | 0.3846 | 0.1989 | 0.0318 | 3.86E-10 | 2.44E-05 | 39.1 |
| rs1209384 | 1 | 43765089 | G | A | 0.6122 | -0.2558 | 0.0313 | 2.85E-16 | 4.19E-05 | 66.8 |
| rs12136922 | 1 | 67007389 | A | G | 0.4949 | 0.2027 | 0.0304 | 2.69E-11 | 2.93E-05 | 44.5 |
| rs12137056 | 1 | 28707612 | T | C | 0.3091 | -0.2327 | 0.0328 | 1.29E-12 | 2.84E-05 | 50.3 |
| rs12153395 | 5 | 179411477 | A | G | 0.1147 | -0.3303 | 0.0486 | 1.07E-11 | 1.24E-05 | 46.2 |
| rs12255372 | 10 | 114808902 | T | G | 0.2883 | 0.2358 | 0.0335 | 1.94E-12 | 2.68E-05 | 49.5 |
| rs12258967 | 10 | 18727959 | G | C | 0.2953 | -0.6327 | 0.0337 | 1.08E-78 | 0.000193639 | 352.5 |
| rs12264186 | 10 | 32289986 | T | C | 0.1871 | 0.2135 | 0.0387 | 3.58E-08 | 1.22E-05 | 30.4 |
| rs12426261 | 12 | 50573037 | G | A | 0.6208 | -0.3775 | 0.0309 | 2.31E-34 | 9.28E-05 | 149.3 |
| rs12464602 | 2 | 43397614 | A | G | 0.6208 | -0.2437 | 0.0315 | 1.02E-14 | 3.72E-05 | 59.9 |
| rs12473915 | 2 | 182987704 | A | G | 0.2017 | -0.295 | 0.0375 | 3.42E-15 | 2.63E-05 | 61.9 |
| rs12509595 | 4 | 81182554 | C | T | 0.2923 | 0.8367 | 0.0334 | 2.55E-138 | 0.0003427 | 627.5 |
| rs12511987 | 4 | 46595623 | G | T | 0.1774 | 0.2329 | 0.0399 | 5.39E-09 | 1.31E-05 | 34.1 |
| rs12596630 | 16 | 2065666 | T | C | 0.0903 | 0.4278 | 0.0547 | 5.01E-15 | 1.33E-05 | 61.2 |
| rs12610654 | 19 | 5006598 | G | A | 0.3436 | -0.2315 | 0.032 | 4.41E-13 | 3.12E-05 | 52.3 |
| rs12627651 | 21 | 44760603 | A | G | 0.2872 | 0.3498 | 0.0341 | 1.02E-24 | 5.69E-05 | 105.2 |
| rs12637573 | 3 | 121682388 | G | A | 0.5282 | 0.1731 | 0.0302 | 9.95E-09 | 2.16E-05 | 32.9 |
| rs12643599 | 4 | 156639846 | G | A | 0.3605 | -0.3134 | 0.0313 | 1.23E-23 | 6.10E-05 | 100.3 |
| rs12656497 | 5 | 32831939 | C | T | 0.5966 | 0.6382 | 0.0307 | 7.14E-96 | 0.000274565 | 432.2 |
| rs12657950 | 5 | 61940569 | T | C | 0.0744 | 0.455 | 0.059 | 1.27E-14 | 1.08E-05 | 59.5 |
| rs12661036 | 6 | 163737476 | C | T | 0.225 | 0.2104 | 0.0374 | 1.82E-08 | 1.46E-05 | 31.6 |
| rs12668436 | 7 | 47548893 | C | T | 0.2459 | 0.2151 | 0.035 | 7.88E-10 | 1.85E-05 | 37.8 |
| rs12693982 | 2 | 204085635 | T | C | 0.4024 | 0.2575 | 0.0309 | 7.49E-17 | 4.41E-05 | 69.4 |
| rs12694277 | 2 | 213188795 | C | T | 0.7054 | 0.2018 | 0.0335 | 1.80E-09 | 1.99E-05 | 36.3 |
| rs12731646 | 1 | 169090660 | T | C | 0.409 | -0.189 | 0.0307 | 7.21E-10 | 2.42E-05 | 37.9 |
| rs1275985 | 2 | 26911745 | T | C | 0.6133 | -0.5411 | 0.0308 | 4.73E-69 | 0.000193237 | 308.6 |
| rs12885878 | 14 | 104007555 | G | A | 0.7663 | 0.2291 | 0.0367 | 4.32E-10 | 1.84E-05 | 39.0 |
| rs12906962 | 15 | 95312071 | C | T | 0.324 | 0.2653 | 0.0325 | 3.28E-16 | 3.85E-05 | 66.6 |
| rs1290784 | 3 | 169096900 | T | C | 0.4483 | 0.4124 | 0.0303 | 2.97E-42 | 0.000120952 | 185.2 |
| rs1290933 | 4 | 2668217 | A | C | 0.6919 | -0.2847 | 0.0327 | 3.17E-18 | 4.27E-05 | 75.8 |
| rs12926550 | 16 | 81510155 | A | G | 0.3156 | -0.2548 | 0.0324 | 3.43E-15 | 3.53E-05 | 61.8 |
| rs1293969 | 6 | 151959945 | C | T | 0.2516 | 0.1988 | 0.0347 | 1.03E-08 | 1.63E-05 | 32.8 |
| rs12978472 | 19 | 7257990 | G | C | 0.1241 | -0.845 | 0.049 | 1.23E-66 | 8.53E-05 | 297.4 |
| rs13016772 | 2 | 55779476 | T | C | 0.7651 | 0.2522 | 0.0355 | 1.23E-12 | 2.39E-05 | 50.5 |
| rs13091418 | 3 | 185329756 | G | C | 0.3341 | 0.2234 | 0.0325 | 6.15E-12 | 2.78E-05 | 47.2 |
| rs13107261 | 4 | 63768826 | A | G | 0.3687 | -0.1778 | 0.0314 | 1.57E-08 | 1.97E-05 | 32.1 |
| rs13107325 | 4 | 103188709 | T | C | 0.0739 | -0.9086 | 0.0592 | 4.22E-53 | 4.26E-05 | 235.6 |
| rs13149209 | 4 | 89750668 | C | T | 0.2227 | -0.281 | 0.0367 | 1.97E-14 | 2.68E-05 | 58.6 |
| rs13253358 | 8 | 68920135 | T | C | 0.2979 | 0.2127 | 0.033 | 1.13E-10 | 2.29E-05 | 41.5 |
| rs1332813 | 9 | 9350706 | C | T | 0.6486 | -0.2203 | 0.0314 | 2.32E-12 | 2.96E-05 | 49.2 |
| rs13358657 | 5 | 157938070 | G | A | 0.1332 | 0.388 | 0.0445 | 2.95E-18 | 2.32E-05 | 76.0 |
| rs13412750 | 2 | 191634958 | A | G | 0.2708 | -0.2889 | 0.0341 | 2.32E-17 | 3.74E-05 | 71.8 |
| rs13420463 | 2 | 37517566 | G | A | 0.2266 | -0.3143 | 0.036 | 2.72E-18 | 3.53E-05 | 76.2 |
| rs1375564 | 3 | 85656311 | T | C | 0.6395 | 0.2579 | 0.0315 | 2.84E-16 | 4.08E-05 | 67.0 |
| rs1382472 | 11 | 27273967 | A | G | 0.4041 | -0.1917 | 0.0307 | 4.47E-10 | 2.48E-05 | 39.0 |
| rs139354822 | 2 | 242344695 | C | T | 0.0296 | -0.6115 | 0.0975 | 3.51E-10 | 2.98E-06 | 39.3 |
| rs1408945 | 1 | 42364877 | T | G | 0.4243 | -0.3196 | 0.0304 | 8.33E-26 | 7.13E-05 | 110.5 |
| rs141958336 | 19 | 2165383 | A | G | 0.043 | 0.7807 | 0.078 | 1.36E-23 | 1.09E-05 | 100.2 |
| rs1422279 | 5 | 122470209 | T | C | 0.3864 | 0.331 | 0.0309 | 1.05E-26 | 7.18E-05 | 114.7 |
| rs1433121 | 19 | 32591878 | T | C | 0.6906 | -0.228 | 0.0326 | 2.66E-12 | 2.76E-05 | 48.9 |
| rs1436138 | 17 | 75316880 | G | A | 0.3633 | -0.3119 | 0.0315 | 4.73E-23 | 5.99E-05 | 98.0 |
| rs1437649 | 18 | 48132646 | A | G | 0.2345 | -0.2189 | 0.0357 | 8.57E-10 | 1.78E-05 | 37.6 |
| rs146550789 | 16 | 66781040 | C | T | 0.0417 | 0.4824 | 0.0778 | 5.64E-10 | 4.06E-06 | 38.4 |
| rs148140538 | 22 | 50228044 | T | C | 0.0808 | -0.3252 | 0.0562 | 7.39E-09 | 6.57E-06 | 33.5 |
| rs148401029 | 8 | 81386066 | A | C | 0.0352 | -0.4623 | 0.0848 | 4.97E-08 | 2.66E-06 | 29.7 |
| rs1493132 | 4 | 108861082 | C | T | 0.3397 | 0.1766 | 0.0318 | 2.73E-08 | 1.83E-05 | 30.8 |
| rs1544861 | 11 | 10679441 | C | T | 0.6605 | -0.1969 | 0.0318 | 5.66E-10 | 2.27E-05 | 38.3 |
| rs1551355 | 17 | 30032420 | T | C | 0.2334 | 0.2098 | 0.0356 | 3.89E-09 | 1.64E-05 | 34.7 |
| rs1565440 | 1 | 243387788 | A | G | 0.3752 | 0.1746 | 0.0311 | 1.94E-08 | 1.95E-05 | 31.5 |
| rs1575290 | 6 | 7715689 | T | C | 0.4733 | 0.1973 | 0.0301 | 5.59E-11 | 2.83E-05 | 43.0 |
| rs1623474 | 10 | 18471794 | T | C | 0.3303 | 0.3827 | 0.0321 | 7.66E-33 | 8.30E-05 | 142.1 |
| rs1630736 | 6 | 12295987 | T | C | 0.465 | -0.1706 | 0.0309 | 3.52E-08 | 2.00E-05 | 30.5 |
| rs1664781 | 5 | 53276301 | A | G | 0.6925 | 0.2643 | 0.0326 | 5.69E-16 | 3.69E-05 | 65.7 |
| rs17010957 | 4 | 86719165 | C | T | 0.1463 | 0.534 | 0.043 | 1.78E-35 | 5.08E-05 | 154.2 |
| rs17035181 | 4 | 157678511 | G | T | 0.1448 | -0.3074 | 0.0429 | 7.61E-13 | 1.68E-05 | 51.3 |
| rs17080102 | 6 | 151004770 | C | G | 0.0694 | -0.8085 | 0.0594 | 3.52E-42 | 3.16E-05 | 185.3 |
| rs17245822 | 13 | 73131694 | C | A | 0.3733 | 0.1899 | 0.0312 | 1.15E-09 | 2.29E-05 | 37.0 |
| rs17249754 | 12 | 90060586 | A | G | 0.1683 | -0.8446 | 0.0403 | 1.25E-97 | 0.000162305 | 439.2 |
| rs17257081 | 2 | 135630498 | G | A | 0.1935 | -0.2274 | 0.0392 | 6.35E-09 | 1.39E-05 | 33.7 |
| rs1745417 | 1 | 228204279 | T | C | 0.5201 | 0.2871 | 0.0301 | 1.59E-21 | 5.99E-05 | 91.0 |
| rs17562391 | 14 | 100133250 | T | C | 0.4186 | 0.1967 | 0.0306 | 1.35E-10 | 2.65E-05 | 41.3 |
| rs17608766 | 17 | 45013271 | C | T | 0.1445 | 0.6903 | 0.0433 | 2.48E-57 | 8.29E-05 | 254.2 |
| rs177551 | 11 | 16930289 | A | C | 0.1344 | 0.373 | 0.0442 | 3.47E-17 | 2.19E-05 | 71.2 |
| rs17760259 | 2 | 19744462 | C | T | 0.4276 | 0.2654 | 0.0304 | 2.25E-18 | 4.92E-05 | 76.2 |
| rs17762 | 11 | 22492454 | A | G | 0.0777 | 0.4117 | 0.0571 | 5.60E-13 | 9.83E-06 | 52.0 |
| rs17812022 | 20 | 19007099 | T | C | 0.0958 | -0.3613 | 0.0525 | 5.65E-12 | 1.08E-05 | 47.4 |
| rs1786345 | 8 | 101674751 | C | A | 0.4338 | -0.2073 | 0.0307 | 1.34E-11 | 2.96E-05 | 45.6 |
| rs1814951 | 4 | 111408718 | A | G | 0.8785 | -0.3231 | 0.0466 | 3.91E-12 | 1.35E-05 | 48.1 |
| rs1821002 | 8 | 10640065 | G | C | 0.5892 | -0.3794 | 0.0307 | 5.19E-35 | 9.76E-05 | 152.7 |
| rs1848994 | 19 | 22111366 | A | G | 0.2828 | 0.2012 | 0.0334 | 1.79E-09 | 1.94E-05 | 36.3 |
| rs1871190 | 5 | 97953719 | T | G | 0.3349 | 0.1954 | 0.0324 | 1.66E-09 | 2.14E-05 | 36.4 |
| rs1882961 | 21 | 16556367 | T | C | 0.3087 | 0.2443 | 0.0326 | 6.69E-14 | 3.16E-05 | 56.2 |
| rs1889785 | 1 | 16348729 | A | G | 0.4552 | 0.1782 | 0.0304 | 4.35E-09 | 2.25E-05 | 34.4 |
| rs189267552 | 3 | 20073193 | A | T | 0.0132 | -0.8664 | 0.139 | 4.55E-10 | 1.34E-06 | 38.9 |
| rs1896326 | 12 | 115342956 | A | G | 0.2291 | -0.2797 | 0.0371 | 4.40E-14 | 2.65E-05 | 56.8 |
| rs1906672 | 8 | 38130025 | A | G | 0.2319 | 0.2966 | 0.0358 | 1.20E-16 | 3.23E-05 | 68.6 |
| rs1957563 | 5 | 157474590 | T | C | 0.265 | 0.3629 | 0.0342 | 2.32E-26 | 5.79E-05 | 112.6 |
| rs1984195 | 6 | 79657391 | A | G | 0.4887 | 0.2409 | 0.0303 | 1.77E-15 | 4.17E-05 | 63.2 |
| rs2014408 | 11 | 16365282 | T | C | 0.2087 | 0.5169 | 0.0373 | 1.26E-43 | 8.37E-05 | 192.0 |
| rs2046341 | 15 | 86040872 | A | G | 0.1921 | -0.2542 | 0.0382 | 2.74E-11 | 1.81E-05 | 44.3 |
| rs2060664 | 16 | 60652439 | C | T | 0.2516 | -0.216 | 0.0345 | 4.06E-10 | 1.95E-05 | 39.2 |
| rs2065498 | 13 | 41893105 | G | T | 0.8294 | 0.2934 | 0.0403 | 3.36E-13 | 1.98E-05 | 53.0 |
| rs2111557 | 3 | 169325621 | T | C | 0.4675 | 0.1764 | 0.0302 | 5.22E-09 | 2.24E-05 | 34.1 |
| rs2126474 | 8 | 76878957 | T | G | 0.4125 | -0.2601 | 0.0306 | 1.87E-17 | 4.62E-05 | 72.3 |
| rs2129869 | 12 | 26457650 | T | A | 0.2222 | 0.2643 | 0.0361 | 2.44E-13 | 2.45E-05 | 53.6 |
| rs2161967 | 2 | 218680529 | G | T | 0.5721 | -0.2836 | 0.0307 | 2.87E-20 | 5.51E-05 | 85.3 |
| rs2177843 | 10 | 75409877 | T | C | 0.1505 | 0.4394 | 0.0432 | 2.80E-24 | 3.49E-05 | 103.5 |
| rs2232460 | 1 | 6659505 | A | G | 0.3343 | -0.2171 | 0.032 | 1.10E-11 | 2.70E-05 | 46.0 |
| rs2236295 | 10 | 64564892 | T | G | 0.3978 | -0.3028 | 0.0309 | 1.04E-22 | 6.07E-05 | 96.0 |
| rs2238787 | 22 | 19976406 | A | G | 0.292 | 0.2552 | 0.0332 | 1.45E-14 | 3.22E-05 | 59.1 |
| rs2249105 | 2 | 65287896 | G | A | 0.3679 | -0.2927 | 0.0313 | 7.63E-21 | 5.37E-05 | 87.4 |
| rs2276153 | 11 | 58407740 | G | C | 0.243 | -0.3296 | 0.0351 | 6.56E-21 | 4.28E-05 | 88.2 |
| rs2283500 | 16 | 4138378 | C | A | 0.1102 | -0.3106 | 0.0481 | 1.08E-10 | 1.08E-05 | 41.7 |
| rs2289124 | 11 | 89224477 | A | G | 0.1673 | -0.308 | 0.0415 | 1.14E-13 | 2.03E-05 | 55.1 |
| rs2291434 | 4 | 38387244 | T | G | 0.5335 | -0.2622 | 0.0303 | 5.10E-18 | 4.92E-05 | 74.9 |
| rs2291516 | 19 | 11508177 | A | G | 0.103 | 0.3708 | 0.0505 | 2.17E-13 | 1.31E-05 | 53.9 |
| rs2327429 | 6 | 134209837 | C | T | 0.2917 | -0.2 | 0.0338 | 3.16E-09 | 1.91E-05 | 35.0 |
| rs2353940 | 4 | 145740898 | C | T | 0.2493 | 0.2075 | 0.0358 | 6.85E-09 | 1.66E-05 | 33.6 |
| rs2354862 | 8 | 64501744 | C | A | 0.3593 | -0.2507 | 0.0317 | 2.42E-15 | 3.80E-05 | 62.5 |
| rs236916 | 11 | 117089628 | A | G | 0.1348 | 0.3166 | 0.0446 | 1.31E-12 | 1.55E-05 | 50.4 |
| rs2384063 | 2 | 25187115 | T | C | 0.7607 | 0.3266 | 0.0357 | 6.33E-20 | 4.02E-05 | 83.7 |
| rs2392929 | 7 | 106414069 | G | T | 0.2027 | 0.7507 | 0.0379 | 1.96E-87 | 0.000167386 | 392.3 |
| rs2423514 | 20 | 10693337 | G | A | 0.4589 | -0.3011 | 0.0302 | 1.77E-23 | 6.52E-05 | 99.4 |
| rs246973 | 5 | 68007803 | T | C | 0.2882 | 0.2479 | 0.0335 | 1.45E-13 | 2.97E-05 | 54.8 |
| rs2470004 | 8 | 120358445 | T | C | 0.8175 | -0.3454 | 0.0392 | 1.28E-18 | 3.06E-05 | 77.6 |
| rs2493134 | 1 | 230849359 | C | T | 0.4071 | 0.3736 | 0.0309 | 1.10E-33 | 9.31E-05 | 146.2 |
| rs2493296 | 1 | 3327032 | T | C | 0.1425 | 0.4183 | 0.0442 | 3.14E-21 | 2.89E-05 | 89.6 |
| rs2498323 | 4 | 3451109 | A | G | 0.098 | 0.3171 | 0.0517 | 8.51E-10 | 8.78E-06 | 37.6 |
| rs2580350 | 2 | 121996007 | A | G | 0.5609 | 0.1769 | 0.0307 | 8.39E-09 | 2.16E-05 | 33.2 |
| rs2589218 | 15 | 96785017 | C | T | 0.2703 | 0.2258 | 0.0339 | 2.54E-11 | 2.31E-05 | 44.4 |
| rs2598 | 20 | 47241618 | G | A | 0.467 | -0.168 | 0.0303 | 2.87E-08 | 2.02E-05 | 30.7 |
| rs2610990 | 4 | 18008232 | G | A | 0.7359 | 0.2903 | 0.0343 | 2.86E-17 | 3.68E-05 | 71.6 |
| rs2627313 | 15 | 81006712 | T | C | 0.4454 | 0.3208 | 0.0303 | 3.55E-26 | 7.31E-05 | 112.1 |
| rs262986 | 3 | 183435713 | A | G | 0.4704 | -0.2371 | 0.0305 | 7.67E-15 | 3.97E-05 | 60.4 |
| rs263532 | 1 | 2164116 | C | T | 0.4245 | -0.1798 | 0.0307 | 4.72E-09 | 2.21E-05 | 34.3 |
| rs2643826 | 3 | 27562988 | T | C | 0.4505 | 0.4473 | 0.0306 | 1.74E-48 | 0.000139639 | 213.7 |
| rs2652812 | 15 | 63406170 | T | C | 0.7544 | -0.2516 | 0.0353 | 1.03E-12 | 2.48E-05 | 50.8 |
| rs2655445 | 6 | 22377623 | A | G | 0.6067 | -0.2018 | 0.0312 | 9.58E-11 | 2.64E-05 | 41.8 |
| rs268263 | 2 | 164954174 | A | T | 0.7498 | 0.5938 | 0.0353 | 1.77E-63 | 0.000140137 | 283.0 |
| rs2689690 | 10 | 95899706 | T | C | 0.3678 | -0.2702 | 0.0316 | 1.15E-17 | 4.49E-05 | 73.1 |
| rs2724377 | 1 | 207974818 | G | A | 0.4697 | -0.1938 | 0.0301 | 1.29E-10 | 2.73E-05 | 41.5 |
| rs2745599 | 6 | 1613686 | G | A | 0.448 | -0.2164 | 0.0317 | 8.96E-12 | 3.04E-05 | 46.6 |
| rs2776037 | 21 | 16317933 | C | T | 0.5849 | 0.1851 | 0.0309 | 2.15E-09 | 2.30E-05 | 35.9 |
| rs2801008 | 20 | 51788718 | G | T | 0.3183 | 0.1876 | 0.0324 | 7.37E-09 | 1.92E-05 | 33.5 |
| rs2815063 | 6 | 39262535 | A | C | 0.1315 | 0.2755 | 0.0458 | 1.76E-09 | 1.09E-05 | 36.2 |
| rs2833834 | 21 | 33814378 | A | C | 0.2765 | 0.2177 | 0.0338 | 1.22E-10 | 2.19E-05 | 41.5 |
| rs28429256 | 15 | 66931617 | A | G | 0.3342 | 0.215 | 0.0325 | 3.89E-11 | 2.57E-05 | 43.8 |
| rs28572357 | 19 | 31867447 | C | A | 0.3977 | 0.2733 | 0.0308 | 6.34E-19 | 4.98E-05 | 78.7 |
| rs28578714 | 22 | 50727921 | C | T | 0.3938 | -0.2066 | 0.0327 | 2.53E-10 | 2.52E-05 | 39.9 |
| rs28650790 | 5 | 55861464 | T | C | 0.1891 | 0.2287 | 0.0387 | 3.30E-09 | 1.41E-05 | 34.9 |
| rs28688791 | 7 | 19039605 | C | T | 0.1982 | 0.3222 | 0.038 | 2.34E-17 | 3.02E-05 | 71.9 |
| rs28866311 | 15 | 41442195 | G | T | 0.4737 | 0.2762 | 0.0302 | 5.45E-20 | 5.51E-05 | 83.6 |
| rs2900568 | 9 | 116689758 | T | C | 0.5184 | -0.1889 | 0.03 | 2.96E-10 | 2.61E-05 | 39.6 |
| rs2904315 | 11 | 48109948 | G | A | 0.6869 | 0.2081 | 0.0325 | 1.58E-10 | 2.33E-05 | 41.0 |
| rs2913920 | 5 | 141726983 | T | C | 0.765 | 0.2418 | 0.0359 | 1.62E-11 | 2.15E-05 | 45.4 |
| rs2957688 | 11 | 10364963 | A | G | 0.4707 | 0.3472 | 0.0304 | 2.74E-30 | 8.58E-05 | 130.4 |
| rs3098186 | 15 | 50810621 | T | C | 0.5156 | -0.2422 | 0.0303 | 1.41E-15 | 4.21E-05 | 63.9 |
| rs3104552 | 9 | 128153616 | C | T | 0.4393 | -0.2449 | 0.0303 | 6.31E-16 | 4.25E-05 | 65.3 |
| rs34025993 | 9 | 123516572 | G | A | 0.586 | -0.223 | 0.0308 | 4.71E-13 | 3.36E-05 | 52.4 |
| rs34072724 | 7 | 130432469 | A | G | 0.4889 | -0.2422 | 0.0303 | 1.37E-15 | 4.21E-05 | 63.9 |
| rs34079867 | 1 | 27407850 | T | C | 0.266 | 0.1992 | 0.0354 | 1.78E-08 | 1.63E-05 | 31.7 |
| rs34130368 | 10 | 48411796 | T | G | 0.117 | -0.3016 | 0.0497 | 1.28E-09 | 1.00E-05 | 36.8 |
| rs34413141 | 18 | 777282 | A | T | 0.1822 | -0.3531 | 0.0393 | 2.47E-19 | 3.18E-05 | 80.7 |
| rs34487963 | 21 | 44838330 | A | C | 0.0185 | -0.8819 | 0.1244 | 1.35E-12 | 2.41E-06 | 50.3 |
| rs34518929 | 19 | 18455444 | A | G | 0.2623 | -0.22 | 0.0345 | 1.79E-10 | 2.08E-05 | 40.7 |
| rs34535756 | 4 | 2246927 | T | C | 0.0394 | 0.478 | 0.0786 | 1.18E-09 | 3.70E-06 | 37.0 |
| rs34917849 | 8 | 95278307 | C | G | 0.1268 | 0.3124 | 0.0454 | 5.97E-12 | 1.38E-05 | 47.3 |
| rs34941092 | 16 | 50550137 | A | G | 0.1498 | -0.3225 | 0.0425 | 3.23E-14 | 1.94E-05 | 57.6 |
| rs35413927 | 14 | 53420358 | G | A | 0.3054 | 0.3002 | 0.0328 | 5.25E-20 | 4.69E-05 | 83.8 |
| rs35444 | 12 | 115552437 | G | A | 0.3862 | -0.4368 | 0.031 | 3.47E-45 | 0.000124243 | 198.5 |
| rs35680304 | 7 | 130973495 | T | C | 0.5929 | 0.2694 | 0.031 | 3.76E-18 | 4.81E-05 | 75.5 |
| rs35783704 | 8 | 105966258 | A | G | 0.1042 | -0.4619 | 0.0507 | 8.81E-20 | 2.05E-05 | 83.0 |
| rs365990 | 14 | 23861811 | G | A | 0.3658 | -0.225 | 0.0312 | 5.95E-13 | 3.19E-05 | 52.0 |
| rs3735533 | 7 | 27245893 | C | T | 0.9257 | 0.91 | 0.0577 | 5.29E-56 | 4.52E-05 | 248.7 |
| rs3754944 | 2 | 231279616 | A | C | 0.5875 | 0.1768 | 0.0308 | 9.30E-09 | 2.11E-05 | 33.0 |
| rs3764400 | 17 | 46123932 | C | T | 0.1365 | -0.3748 | 0.0445 | 3.69E-17 | 2.21E-05 | 70.9 |
| rs3772219 | 3 | 56771251 | C | A | 0.3176 | -0.2733 | 0.0324 | 3.10E-17 | 4.07E-05 | 71.2 |
| rs3807925 | 7 | 18543250 | G | A | 0.3504 | 0.1859 | 0.0319 | 5.39E-09 | 2.04E-05 | 34.0 |
| rs3819532 | 12 | 2436837 | C | T | 0.6087 | 0.1875 | 0.0306 | 9.44E-10 | 2.36E-05 | 37.5 |
| rs3860770 | 5 | 173301427 | A | G | 0.2916 | -0.2663 | 0.0333 | 1.20E-15 | 3.49E-05 | 64.0 |
| rs3918226 | 7 | 150690176 | T | C | 0.0811 | 0.664 | 0.0575 | 8.46E-31 | 2.62E-05 | 133.4 |
| rs3950627 | 16 | 86436343 | A | C | 0.531 | 0.1851 | 0.0308 | 1.82E-09 | 2.37E-05 | 36.1 |
| rs3980686 | 3 | 168697602 | T | G | 0.1075 | -0.4998 | 0.0487 | 1.03E-24 | 2.67E-05 | 105.3 |
| rs404100 | 1 | 25366987 | T | C | 0.4513 | 0.1935 | 0.0303 | 1.68E-10 | 2.67E-05 | 40.8 |
| rs4143175 | 12 | 67782397 | C | T | 0.7591 | -0.2187 | 0.0352 | 5.10E-10 | 1.86E-05 | 38.6 |
| rs42032 | 7 | 92237426 | A | G | 0.2641 | -0.3231 | 0.0345 | 7.39E-21 | 4.50E-05 | 87.7 |
| rs4245599 | 10 | 60365755 | G | A | 0.5416 | 0.1794 | 0.0305 | 4.03E-09 | 2.27E-05 | 34.6 |
| rs4284362 | 10 | 45377839 | A | C | 0.7181 | -0.2256 | 0.0338 | 2.61E-11 | 2.38E-05 | 44.5 |
| rs4408839 | 3 | 153729768 | G | A | 0.2567 | 0.2301 | 0.0345 | 2.42E-11 | 2.24E-05 | 44.5 |
| rs4440615 | 8 | 141057641 | A | G | 0.6321 | -0.2201 | 0.0312 | 1.87E-12 | 3.06E-05 | 49.8 |
| rs4499560 | 3 | 70920485 | T | A | 0.6829 | 0.2199 | 0.0326 | 1.46E-11 | 2.60E-05 | 45.5 |
| rs4511593 | 17 | 7455536 | T | C | 0.6528 | -0.2881 | 0.0318 | 1.28E-19 | 4.91E-05 | 82.1 |
| rs4553000 | 9 | 34223553 | T | C | 0.5141 | -0.2035 | 0.03 | 1.09E-11 | 3.03E-05 | 46.0 |
| rs4577304 | 2 | 73403040 | C | T | 0.4767 | 0.1767 | 0.0302 | 4.99E-09 | 2.25E-05 | 34.2 |
| rs4598218 | 8 | 129483956 | T | C | 0.6158 | 0.1911 | 0.0313 | 1.00E-09 | 2.33E-05 | 37.3 |
| rs4606697 | 15 | 100087596 | A | G | 0.1041 | -0.3196 | 0.0523 | 9.71E-10 | 9.19E-06 | 37.3 |
| rs4651224 | 1 | 184585182 | T | C | 0.4474 | 0.1986 | 0.0306 | 9.00E-11 | 2.75E-05 | 42.1 |
| rs4775769 | 15 | 48939888 | G | T | 0.9055 | 0.4162 | 0.0517 | 7.76E-16 | 1.46E-05 | 64.8 |
| rs4784541 | 16 | 51704452 | C | T | 0.5252 | 0.2015 | 0.0307 | 4.93E-11 | 2.84E-05 | 43.1 |
| rs483071 | 13 | 22294117 | T | C | 0.6248 | 0.2709 | 0.0313 | 5.09E-18 | 4.64E-05 | 74.9 |
| rs4873492 | 8 | 51947549 | T | C | 0.1724 | 0.3431 | 0.0403 | 1.61E-17 | 2.73E-05 | 72.5 |
| rs488834 | 1 | 10767902 | T | C | 0.7645 | -0.3799 | 0.0365 | 2.35E-25 | 5.15E-05 | 108.3 |
| rs4888408 | 16 | 75432824 | A | G | 0.5855 | 0.3653 | 0.0307 | 1.42E-32 | 9.07E-05 | 141.6 |
| rs4894132 | 2 | 180738654 | C | T | 0.2717 | -0.2469 | 0.0342 | 5.51E-13 | 2.72E-05 | 52.1 |
| rs4925159 | 17 | 18185510 | A | G | 0.4246 | 0.2174 | 0.0305 | 9.66E-13 | 3.28E-05 | 50.8 |
| rs4926499 | 1 | 249155909 | C | G | 0.8263 | 0.2965 | 0.0438 | 1.33E-11 | 1.74E-05 | 45.8 |
| rs4932373 | 15 | 91429287 | C | A | 0.3258 | 0.635 | 0.0328 | 2.49E-83 | 0.000217335 | 374.8 |
| rs4952609 | 2 | 40555733 | G | A | 0.2561 | -0.2124 | 0.0347 | 9.60E-10 | 1.88E-05 | 37.5 |
| rs4955575 | 3 | 169534538 | C | A | 0.2539 | -0.2158 | 0.0348 | 5.63E-10 | 1.92E-05 | 38.5 |
| rs4957026 | 5 | 361148 | G | A | 0.6601 | -0.1982 | 0.0323 | 8.12E-10 | 2.23E-05 | 37.7 |
| rs4961293 | 8 | 141812374 | T | C | 0.4513 | 0.2268 | 0.0303 | 7.35E-14 | 3.66E-05 | 56.0 |
| rs4980379 | 11 | 1888614 | T | C | 0.3719 | 0.5764 | 0.032 | 2.47E-72 | 0.000200075 | 324.5 |
| rs5020545 | 4 | 77414988 | T | C | 0.4437 | -0.2179 | 0.0305 | 9.71E-13 | 3.33E-05 | 51.0 |
| rs509833 | 6 | 159711515 | G | A | 0.8614 | -0.329 | 0.044 | 7.08E-14 | 1.76E-05 | 55.9 |
| rs55732192 | 2 | 162278233 | T | G | 0.0947 | -0.3358 | 0.0521 | 1.15E-10 | 9.40E-06 | 41.5 |
| rs55924432 | 4 | 26812737 | T | C | 0.401 | 0.2651 | 0.0317 | 5.70E-17 | 4.43E-05 | 69.9 |
| rs55944332 | 2 | 145726621 | G | A | 0.2368 | 0.2613 | 0.0355 | 1.79E-13 | 2.58E-05 | 54.2 |
| rs56288724 | 17 | 60767135 | G | A | 0.4169 | 0.2178 | 0.031 | 2.01E-12 | 3.17E-05 | 49.4 |
| rs56407827 | 18 | 42179819 | T | C | 0.2687 | 0.3603 | 0.034 | 2.78E-26 | 5.83E-05 | 112.3 |
| rs57140819 | 14 | 68018247 | G | C | 0.1732 | -0.2415 | 0.0398 | 1.30E-09 | 1.39E-05 | 36.8 |
| rs571689 | 19 | 49207554 | T | C | 0.5196 | 0.228 | 0.0304 | 6.77E-14 | 3.71E-05 | 56.3 |
| rs573455 | 11 | 117267884 | G | A | 0.539 | -0.1994 | 0.0303 | 4.77E-11 | 2.84E-05 | 43.3 |
| rs5742643 | 12 | 102837863 | C | T | 0.7513 | 0.2233 | 0.0349 | 1.53E-10 | 2.02E-05 | 40.9 |
| rs57866767 | 10 | 96023077 | C | T | 0.4322 | -0.4501 | 0.0304 | 1.14E-49 | 0.000142017 | 219.2 |
| rs57946343 | 10 | 63499951 | C | T | 0.1473 | -0.716 | 0.0426 | 2.10E-63 | 9.37E-05 | 282.5 |
| rs59980837 | 1 | 115827266 | T | G | 0.0178 | 1.0997 | 0.1163 | 3.32E-21 | 4.13E-06 | 89.4 |
| rs60191654 | 9 | 753648 | G | A | 0.1882 | 0.2382 | 0.0385 | 5.88E-10 | 1.54E-05 | 38.3 |
| rs6026578 | 20 | 57463472 | G | C | 0.6269 | 0.185 | 0.0316 | 4.59E-09 | 2.12E-05 | 34.3 |
| rs6026744 | 20 | 57742388 | T | A | 0.1229 | 0.7131 | 0.0461 | 7.00E-54 | 6.81E-05 | 239.3 |
| rs6029756 | 20 | 40266681 | A | G | 0.3225 | -0.2712 | 0.033 | 1.88E-16 | 3.90E-05 | 67.5 |
| rs60444686 | 10 | 115717311 | A | G | 0.0406 | 0.5905 | 0.0782 | 4.42E-14 | 5.86E-06 | 57.0 |
| rs604723 | 11 | 100610546 | C | T | 0.7244 | 0.655 | 0.0339 | 2.55E-83 | 0.000196757 | 373.3 |
| rs6054139 | 20 | 6327810 | A | G | 0.606 | 0.2094 | 0.0306 | 8.23E-12 | 2.95E-05 | 46.8 |
| rs6058088 | 20 | 30139886 | G | T | 0.1561 | -0.2832 | 0.0417 | 1.14E-11 | 1.60E-05 | 46.1 |
| rs6062324 | 20 | 62446351 | A | G | 0.2364 | -0.3294 | 0.0363 | 1.18E-19 | 3.92E-05 | 82.3 |
| rs6078093 | 20 | 11168669 | A | G | 0.428 | -0.1849 | 0.0304 | 1.20E-09 | 2.39E-05 | 37.0 |
| rs6090907 | 20 | 47410231 | A | G | 0.147 | -0.3854 | 0.0425 | 1.29E-19 | 2.72E-05 | 82.2 |
| rs6108787 | 20 | 10967214 | G | T | 0.4704 | 0.4274 | 0.03 | 5.38E-46 | 0.000133485 | 203.0 |
| rs61772592 | 1 | 56979681 | G | A | 0.1255 | 0.3181 | 0.0455 | 2.86E-12 | 1.42E-05 | 48.9 |
| rs61917655 | 12 | 48210787 | T | C | 0.1014 | 0.3427 | 0.0514 | 2.68E-11 | 1.07E-05 | 44.5 |
| rs62047964 | 16 | 70729954 | T | C | 0.0622 | 0.5115 | 0.0686 | 9.29E-14 | 8.56E-06 | 55.6 |
| rs62076622 | 17 | 61090958 | G | A | 0.1987 | -0.2363 | 0.0377 | 3.79E-10 | 1.65E-05 | 39.3 |
| rs62082230 | 18 | 22676071 | A | T | 0.2773 | -0.1884 | 0.0345 | 4.69E-08 | 1.58E-05 | 29.8 |
| rs62170470 | 2 | 146989797 | C | T | 0.3983 | -0.1972 | 0.0321 | 7.68E-10 | 2.39E-05 | 37.7 |
| rs62309747 | 4 | 48713862 | A | G | 0.4734 | -0.2244 | 0.0304 | 1.59E-13 | 3.59E-05 | 54.5 |
| rs62512914 | 8 | 82828857 | G | A | 0.4152 | -0.2066 | 0.0306 | 1.42E-11 | 2.92E-05 | 45.6 |
| rs629864 | 11 | 100699139 | T | C | 0.6497 | -0.1868 | 0.0319 | 4.69E-09 | 2.06E-05 | 34.3 |
| rs6438857 | 3 | 124557643 | C | T | 0.4226 | -0.2736 | 0.0305 | 3.13E-19 | 5.18E-05 | 80.5 |
| rs6445583 | 3 | 53562894 | A | G | 0.7465 | 0.2774 | 0.0349 | 1.90E-15 | 3.16E-05 | 63.2 |
| rs6452769 | 5 | 87389027 | A | G | 0.2053 | -0.3143 | 0.0377 | 7.82E-17 | 2.99E-05 | 69.5 |
| rs6490019 | 12 | 115920472 | G | A | 0.6204 | 0.2897 | 0.0309 | 6.61E-21 | 5.46E-05 | 87.9 |
| rs6504213 | 17 | 62381714 | C | T | 0.5818 | 0.2982 | 0.0312 | 1.25E-21 | 5.87E-05 | 91.3 |
| rs6540119 | 16 | 87984477 | T | A | 0.666 | -0.2016 | 0.0322 | 3.93E-10 | 2.30E-05 | 39.2 |
| rs6558535 | 8 | 1710632 | C | T | 0.7146 | 0.216 | 0.0336 | 1.33E-10 | 2.23E-05 | 41.3 |
| rs6562778 | 13 | 74223828 | G | A | 0.5411 | -0.178 | 0.0304 | 4.95E-09 | 2.25E-05 | 34.3 |
| rs658780 | 1 | 78555928 | G | T | 0.2553 | 0.2028 | 0.0347 | 5.29E-09 | 1.71E-05 | 34.2 |
| rs665445 | 18 | 51842682 | A | C | 0.2794 | -0.1909 | 0.0334 | 1.15E-08 | 1.74E-05 | 32.7 |
| rs66864335 | 11 | 65390803 | A | G | 0.2211 | -0.3963 | 0.0365 | 1.79E-27 | 5.36E-05 | 117.9 |
| rs6699618 | 1 | 11881441 | G | C | 0.1599 | -0.9115 | 0.041 | 1.68E-109 | 0.000175273 | 494.2 |
| rs6731373 | 2 | 68503044 | A | G | 0.3492 | 0.1913 | 0.0326 | 4.18E-09 | 2.07E-05 | 34.4 |
| rs6737318 | 2 | 114083120 | G | A | 0.2218 | -0.2348 | 0.0364 | 1.13E-10 | 1.90E-05 | 41.6 |
| rs67617547 | 7 | 90297177 | G | C | 0.3303 | -0.1799 | 0.0322 | 2.39E-08 | 1.82E-05 | 31.2 |
| rs6771917 | 3 | 48108442 | C | T | 0.7523 | 0.3793 | 0.0355 | 1.39E-26 | 5.62E-05 | 114.2 |
| rs6788907 | 3 | 158212823 | A | G | 0.2682 | 0.221 | 0.0339 | 7.31E-11 | 2.20E-05 | 42.5 |
| rs6788984 | 3 | 41107173 | G | A | 0.1437 | -0.2999 | 0.0432 | 3.81E-12 | 1.57E-05 | 48.2 |
| rs68085857 | 1 | 217737629 | T | C | 0.234 | 0.274 | 0.0357 | 1.68E-14 | 2.79E-05 | 58.9 |
| rs68115553 | 3 | 27704702 | G | A | 0.0199 | 0.6445 | 0.1143 | 1.74E-08 | 1.64E-06 | 31.8 |
| rs6823199 | 4 | 83925895 | C | T | 0.2562 | -0.2094 | 0.0348 | 1.72E-09 | 1.82E-05 | 36.2 |
| rs6870654 | 5 | 63831964 | C | T | 0.2546 | -0.2136 | 0.0347 | 7.58E-10 | 1.90E-05 | 37.9 |
| rs6892983 | 5 | 127845030 | A | C | 0.4022 | 0.3427 | 0.0307 | 7.11E-29 | 7.91E-05 | 124.6 |
| rs6921291 | 6 | 97066242 | T | C | 0.1907 | 0.3575 | 0.0385 | 1.58E-20 | 3.51E-05 | 86.2 |
| rs6961048 | 7 | 27328187 | G | C | 0.104 | 0.5304 | 0.0497 | 1.43E-26 | 2.80E-05 | 113.9 |
| rs6978112 | 7 | 1966841 | T | C | 0.4112 | 0.2286 | 0.0309 | 1.34E-13 | 3.50E-05 | 54.7 |
| rs7012866 | 8 | 135616959 | G | T | 0.5009 | 0.2325 | 0.0301 | 1.21E-14 | 3.94E-05 | 59.7 |
| rs702395 | 5 | 140086677 | T | C | 0.4369 | 0.2318 | 0.0305 | 3.24E-14 | 3.75E-05 | 57.8 |
| rs7045409 | 9 | 95201540 | A | T | 0.3669 | -0.1862 | 0.0313 | 2.55E-09 | 2.17E-05 | 35.4 |
| rs7093894 | 10 | 124234880 | A | C | 0.1512 | 0.236 | 0.0427 | 3.16E-08 | 1.03E-05 | 30.5 |
| rs7107356 | 11 | 47676170 | G | A | 0.5041 | 0.4598 | 0.0301 | 1.63E-52 | 0.000153994 | 233.3 |
| rs7134440 | 12 | 53450097 | T | C | 0.0822 | 0.4788 | 0.0562 | 1.58E-17 | 1.45E-05 | 72.6 |
| rs7134677 | 12 | 54441498 | T | C | 0.2978 | -0.3851 | 0.0332 | 4.46E-31 | 7.43E-05 | 134.5 |
| rs71421551 | 2 | 177053298 | C | G | 0.2885 | 0.2374 | 0.0333 | 1.05E-12 | 2.75E-05 | 50.8 |
| rs7154723 | 14 | 98590629 | A | G | 0.385 | 0.253 | 0.0309 | 2.72E-16 | 4.19E-05 | 67.0 |
| rs7186298 | 16 | 21088031 | T | C | 0.4295 | -0.2315 | 0.0302 | 1.88E-14 | 3.80E-05 | 58.8 |
| rs7213273 | 17 | 43155914 | A | G | 0.655 | -0.4 | 0.0315 | 6.24E-37 | 9.62E-05 | 161.2 |
| rs7218708 | 17 | 19926836 | G | A | 0.5169 | 0.1781 | 0.0303 | 4.38E-09 | 2.28E-05 | 34.5 |
| rs7245140 | 18 | 43095231 | C | T | 0.1802 | 0.3367 | 0.0391 | 7.67E-18 | 2.89E-05 | 74.2 |
| rs7255933 | 19 | 45766729 | A | G | 0.2574 | 0.2306 | 0.0345 | 2.44E-11 | 2.25E-05 | 44.7 |
| rs72683923 | 14 | 50735947 | C | T | 0.0212 | -0.9587 | 0.1101 | 3.08E-18 | 4.15E-06 | 75.8 |
| rs72719160 | 4 | 144051276 | T | A | 0.3171 | 0.2243 | 0.0324 | 4.34E-12 | 2.74E-05 | 47.9 |
| rs72742507 | 1 | 221265336 | T | C | 0.2999 | -0.2053 | 0.0328 | 3.80E-10 | 2.17E-05 | 39.2 |
| rs72778133 | 16 | 3578718 | C | T | 0.1422 | 0.2417 | 0.0443 | 4.97E-08 | 9.59E-06 | 29.8 |
| rs7278003 | 21 | 44966069 | C | T | 0.5622 | 0.1876 | 0.0304 | 6.63E-10 | 2.47E-05 | 38.1 |
| rs72847885 | 2 | 86326717 | G | A | 0.337 | -0.2413 | 0.0318 | 3.08E-14 | 3.40E-05 | 57.6 |
| rs72931748 | 11 | 69825414 | G | A | 0.0985 | -0.3967 | 0.0529 | 6.40E-14 | 1.32E-05 | 56.2 |
| rs73040775 | 19 | 30351998 | T | C | 0.0641 | -0.3387 | 0.0619 | 4.49E-08 | 4.74E-06 | 29.9 |
| rs73046792 | 19 | 49605705 | A | G | 0.1588 | -0.3554 | 0.0426 | 7.23E-17 | 2.45E-05 | 69.6 |
| rs73049928 | 7 | 4669949 | G | A | 0.1939 | 0.2382 | 0.0392 | 1.20E-09 | 1.52E-05 | 36.9 |
| rs7306710 | 12 | 66376091 | C | T | 0.519 | 0.2429 | 0.0303 | 1.02E-15 | 4.24E-05 | 64.3 |
| rs73075659 | 12 | 20373541 | G | A | 0.3346 | -0.3962 | 0.0321 | 5.52E-35 | 8.95E-05 | 152.3 |
| rs73103937 | 5 | 66280577 | C | T | 0.268 | -0.2051 | 0.0344 | 2.36E-09 | 1.84E-05 | 35.5 |
| rs7331680 | 13 | 115000650 | T | G | 0.1491 | 0.4101 | 0.0423 | 3.35E-22 | 3.15E-05 | 94.0 |
| rs73727605 | 7 | 149474622 | A | G | 0.0663 | 0.3616 | 0.0623 | 6.60E-09 | 5.51E-06 | 33.7 |
| rs73855810 | 4 | 148383424 | A | G | 0.1406 | 0.2732 | 0.0434 | 3.04E-10 | 1.26E-05 | 39.6 |
| rs7395791 | 11 | 69262916 | A | G | 0.4419 | -0.2162 | 0.0308 | 2.19E-12 | 3.21E-05 | 49.3 |
| rs74048190 | 11 | 2114221 | C | T | 0.0478 | 0.4404 | 0.0757 | 6.07E-09 | 4.07E-06 | 33.8 |
| rs740746 | 10 | 115792787 | A | G | 0.7318 | 0.4557 | 0.0342 | 1.42E-40 | 9.20E-05 | 177.5 |
| rs743395 | 3 | 37598382 | T | C | 0.3834 | 0.2597 | 0.0317 | 2.55E-16 | 4.19E-05 | 67.1 |
| rs7439567 | 4 | 138464842 | T | C | 0.4106 | 0.2537 | 0.0309 | 2.31E-16 | 4.31E-05 | 67.4 |
| rs74538877 | 11 | 67976593 | C | G | 0.0557 | -0.3855 | 0.0707 | 4.97E-08 | 4.13E-06 | 29.7 |
| rs7491248 | 13 | 47180671 | A | G | 0.2239 | 0.2163 | 0.0362 | 2.37E-09 | 1.64E-05 | 35.7 |
| rs7493678 | 14 | 39400917 | T | A | 0.3486 | 0.189 | 0.0316 | 2.31E-09 | 2.14E-05 | 35.8 |
| rs75016974 | 14 | 100197940 | T | C | 0.1423 | -0.2513 | 0.0439 | 1.05E-08 | 1.06E-05 | 32.8 |
| rs7514579 | 1 | 94051350 | C | A | 0.2288 | -0.2243 | 0.0361 | 5.45E-10 | 1.80E-05 | 38.6 |
| rs75461554 | 1 | 15810172 | T | C | 0.2007 | -0.3016 | 0.0377 | 1.18E-15 | 2.71E-05 | 64.0 |
| rs7555285 | 1 | 209970355 | C | G | 0.8011 | 0.2294 | 0.0376 | 1.05E-09 | 1.57E-05 | 37.2 |
| rs75672964 | 7 | 131321010 | T | C | 0.0418 | 0.5885 | 0.0839 | 2.35E-12 | 5.20E-06 | 49.2 |
| rs75961402 | 13 | 56398286 | A | G | 0.1534 | 0.2659 | 0.0418 | 1.95E-10 | 1.39E-05 | 40.5 |
| rs7615099 | 3 | 53143901 | G | A | 0.3325 | -0.1891 | 0.0321 | 3.90E-09 | 2.03E-05 | 34.7 |
| rs7618284 | 3 | 66422246 | C | G | 0.3394 | -0.1891 | 0.0331 | 1.10E-08 | 1.93E-05 | 32.6 |
| rs76443575 | 5 | 96211594 | C | G | 0.0359 | -0.5233 | 0.0816 | 1.40E-10 | 3.76E-06 | 41.1 |
| rs76452347 | 9 | 35906471 | T | C | 0.205 | -0.2974 | 0.0397 | 7.13E-14 | 2.41E-05 | 56.1 |
| rs76719272 | 1 | 156129796 | T | C | 0.1312 | -0.2738 | 0.0461 | 2.97E-09 | 1.06E-05 | 35.3 |
| rs7683728 | 4 | 156402654 | T | C | 0.5312 | -0.3654 | 0.0304 | 2.43E-33 | 9.50E-05 | 144.5 |
| rs77032376 | 15 | 90010780 | T | C | 0.1485 | -0.2727 | 0.043 | 2.35E-10 | 1.34E-05 | 40.2 |
| rs7703560 | 5 | 67678506 | G | A | 0.2998 | 0.2246 | 0.0333 | 1.51E-11 | 2.52E-05 | 45.5 |
| rs7722243 | 5 | 50818437 | A | G | 0.5043 | -0.2048 | 0.0302 | 1.21E-11 | 3.03E-05 | 46.0 |
| rs7725413 | 5 | 15695987 | T | C | 0.7699 | -0.1985 | 0.0359 | 3.07E-08 | 1.43E-05 | 30.6 |
| rs77375686 | 8 | 26043622 | G | A | 0.1117 | 0.3467 | 0.0485 | 8.38E-13 | 1.34E-05 | 51.1 |
| rs7744902 | 6 | 166176722 | A | G | 0.0766 | -0.4088 | 0.0593 | 5.64E-12 | 8.87E-06 | 47.5 |
| rs7763558 | 6 | 43349215 | A | G | 0.3241 | 0.3363 | 0.0321 | 1.17E-25 | 6.35E-05 | 109.8 |
| rs7765526 | 6 | 147713764 | G | A | 0.5367 | -0.201 | 0.0307 | 5.88E-11 | 2.81E-05 | 42.9 |
| rs778124 | 1 | 56606206 | A | G | 0.3736 | 0.2965 | 0.0311 | 1.45E-21 | 5.62E-05 | 90.9 |
| rs77924615 | 16 | 20392332 | A | G | 0.1986 | -0.4081 | 0.039 | 1.12E-25 | 4.60E-05 | 109.5 |
| rs7821832 | 8 | 25889446 | G | T | 0.2553 | -0.4222 | 0.0348 | 6.67E-34 | 7.39E-05 | 147.2 |
| rs7830689 | 8 | 92777060 | A | G | 0.4221 | 0.1766 | 0.0306 | 8.07E-09 | 2.14E-05 | 33.3 |
| rs78474310 | 13 | 73826901 | G | A | 0.0448 | 0.4699 | 0.0734 | 1.51E-10 | 4.63E-06 | 41.0 |
| rs7854147 | 9 | 125863350 | G | A | 0.123 | -0.3056 | 0.0461 | 3.29E-11 | 1.25E-05 | 43.9 |
| rs78648104 | 6 | 50683009 | C | T | 0.0925 | 0.4287 | 0.0541 | 2.36E-15 | 1.39E-05 | 62.8 |
| rs786923 | 1 | 89242954 | T | C | 0.6239 | -0.3082 | 0.031 | 2.82E-23 | 6.12E-05 | 98.8 |
| rs78998485 | 12 | 434755 | G | C | 0.2557 | 0.2449 | 0.0346 | 1.48E-12 | 2.52E-05 | 50.1 |
| rs79069610 | 8 | 105921209 | C | T | 0.05 | 0.4005 | 0.0727 | 3.68E-08 | 3.81E-06 | 30.3 |
| rs7912283 | 10 | 133773019 | A | G | 0.6468 | -0.2144 | 0.0322 | 2.94E-11 | 2.67E-05 | 44.3 |
| rs7926110 | 11 | 107086143 | G | T | 0.3267 | -0.2603 | 0.0321 | 5.71E-16 | 3.82E-05 | 65.8 |
| rs79384779 | 20 | 31214944 | T | C | 0.1512 | 0.3179 | 0.0428 | 1.08E-13 | 1.87E-05 | 55.2 |
| rs79539362 | 3 | 154680449 | C | T | 0.1008 | -0.4003 | 0.0504 | 2.09E-15 | 1.51E-05 | 63.1 |
| rs7963801 | 12 | 79685226 | C | T | 0.5779 | 0.2362 | 0.0311 | 2.87E-14 | 3.71E-05 | 57.7 |
| rs79782817 | 6 | 25882678 | T | G | 0.1027 | 0.5324 | 0.0499 | 1.43E-26 | 2.77E-05 | 113.8 |
| rs7980644 | 12 | 79959658 | G | A | 0.8334 | -0.2641 | 0.0404 | 6.30E-11 | 1.57E-05 | 42.7 |
| rs8030856 | 15 | 40314967 | G | C | 0.3953 | 0.1764 | 0.031 | 1.21E-08 | 2.04E-05 | 32.4 |
| rs8044992 | 16 | 24811207 | C | T | 0.2877 | -0.2138 | 0.0331 | 1.07E-10 | 2.26E-05 | 41.7 |
| rs8105174 | 19 | 10347032 | T | C | 0.1818 | -0.2374 | 0.0398 | 2.43E-09 | 1.40E-05 | 35.6 |
| rs8125763 | 20 | 17883531 | A | C | 0.4717 | 0.1761 | 0.0301 | 4.84E-09 | 2.25E-05 | 34.2 |
| rs8142376 | 22 | 32001037 | T | C | 0.491 | 0.1676 | 0.03 | 2.19E-08 | 2.06E-05 | 31.2 |
| rs8180684 | 6 | 143200936 | T | C | 0.2896 | 0.2134 | 0.0335 | 1.80E-10 | 2.20E-05 | 40.6 |
| rs848445 | 7 | 77572461 | C | T | 0.7149 | 0.2025 | 0.0339 | 2.28E-09 | 1.92E-05 | 35.7 |
| rs869396 | 4 | 169688000 | A | C | 0.4659 | -0.2115 | 0.0305 | 4.12E-12 | 3.16E-05 | 48.1 |
| rs871004 | 11 | 28512458 | A | G | 0.3481 | 0.2336 | 0.0317 | 1.65E-13 | 3.25E-05 | 54.3 |
| rs8904 | 14 | 35871217 | A | G | 0.3678 | 0.3061 | 0.0314 | 1.71E-22 | 5.83E-05 | 95.0 |
| rs908951 | 16 | 89697625 | T | C | 0.4378 | -0.2261 | 0.0315 | 7.14E-13 | 3.35E-05 | 51.5 |
| rs927315 | 9 | 4117713 | T | C | 0.4713 | 0.1689 | 0.0303 | 2.44E-08 | 2.04E-05 | 31.1 |
| rs9302885 | 17 | 76799898 | G | A | 0.5548 | -0.2242 | 0.0302 | 1.03E-13 | 3.59E-05 | 55.1 |
| rs9303175 | 17 | 1372987 | G | T | 0.6537 | 0.2048 | 0.0327 | 3.65E-10 | 2.34E-05 | 39.2 |
| rs9327297 | 5 | 122835051 | G | C | 0.3324 | -0.2747 | 0.0319 | 8.07E-18 | 4.34E-05 | 74.2 |
| rs9349379 | 6 | 12903957 | G | A | 0.407 | -0.2664 | 0.0312 | 1.31E-17 | 4.65E-05 | 72.9 |
| rs9361836 | 6 | 82235408 | T | C | 0.3172 | 0.2196 | 0.0324 | 1.25E-11 | 2.63E-05 | 45.9 |
| rs9368222 | 6 | 20686996 | A | C | 0.2688 | 0.2281 | 0.0339 | 1.84E-11 | 2.35E-05 | 45.3 |
| rs9401913 | 6 | 127159982 | A | G | 0.4387 | 0.5202 | 0.0305 | 3.66E-65 | 0.000189101 | 290.9 |
| rs9486916 | 6 | 109013930 | T | C | 0.1979 | 0.2657 | 0.0385 | 5.42E-12 | 2.00E-05 | 47.6 |
| rs9507885 | 13 | 27951090 | T | C | 0.0953 | -0.3208 | 0.0542 | 3.23E-09 | 7.97E-06 | 35.0 |
| rs9508495 | 13 | 30146201 | T | C | 0.7565 | -0.3557 | 0.0353 | 6.34E-24 | 4.94E-05 | 101.5 |
| rs9526707 | 13 | 51489186 | A | G | 0.3216 | -0.2039 | 0.0323 | 2.77E-10 | 2.30E-05 | 39.9 |
| rs9549627 | 13 | 113652369 | A | G | 0.1175 | 0.2846 | 0.05 | 1.25E-08 | 8.87E-06 | 32.4 |
| rs9651825 | 12 | 27159784 | G | A | 0.2705 | 0.2042 | 0.034 | 1.93E-09 | 1.88E-05 | 36.1 |
| rs977184 | 7 | 28650761 | C | T | 0.3748 | 0.184 | 0.0314 | 4.86E-09 | 2.12E-05 | 34.3 |
| rs9848170 | 3 | 11495983 | C | G | 0.597 | 0.3231 | 0.0307 | 7.01E-26 | 7.04E-05 | 110.8 |
| rs9869437 | 3 | 196228360 | A | C | 0.3523 | -0.2001 | 0.0318 | 3.22E-10 | 2.39E-05 | 39.6 |
| rs9876694 | 3 | 141152017 | T | C | 0.0584 | 0.4713 | 0.0651 | 4.64E-13 | 7.61E-06 | 52.4 |
| rs9880098 | 3 | 133949366 | A | G | 0.3946 | 0.3081 | 0.0308 | 1.59E-23 | 6.31E-05 | 100.1 |
| rs9886665 | 9 | 22942770 | C | T | 0.7329 | -0.2048 | 0.0343 | 2.47E-09 | 1.84E-05 | 35.7 |
| rs9897429 | 17 | 47518378 | A | G | 0.52 | 0.2645 | 0.0319 | 1.19E-16 | 4.53E-05 | 68.7 |
| rs9899540 | 17 | 30777924 | T | A | 0.6001 | -0.2011 | 0.0316 | 1.87E-10 | 2.57E-05 | 40.5 |
| rs9918879 | 8 | 77681093 | T | G | 0.103 | -0.2984 | 0.0499 | 2.28E-09 | 8.72E-06 | 35.8 |

**SNP: single nucleotide polymorphism; CHR: chromosome; EAF: effect allele frequency; SE: standard error.**
