# Supplementary material for: Hypertension, antihypertensive drugs, and age at onset of Huntington’s disease
Source: Orphanet J Rare Dis. 2023 May 24;18:125. doi: 10.1186/s13023-023-02734-1 (PMC10207760; doi:10.1186/s13023-023-02734-1)
Supplement: Supplementary file 2 — Supplementary Material 2 [file 13023_2023_2734_MOESM2_ESM.docx]

**Table S2 The characteristics of the selected DBP instrumental variables**

| **SNP** | **Chr** | **Position**  **(GRCh37/hg19)** | **Effect allele** | **Other allele** | **EAF** | **beta** | **SE** | **P-value** | **R^2^** | **F** |
| --- | --- | --- | --- | --- | --- | --- | --- | --- | --- | --- |
| rs1003763 | 21 | 40073020 | G | C | 0.753 | 0.1484 | 0.0203 | 2.75E-13 | 2.62E-05 | 53.4 |
| rs10054208 | 5 | 55688992 | T | C | 0.3617 | 0.1187 | 0.0185 | 1.49E-10 | 2.51E-05 | 41.2 |
| rs10062049 | 5 | 61553881 | T | C | 0.1359 | 0.2208 | 0.0255 | 4.50E-18 | 2.32E-05 | 75.0 |
| rs1006545 | 10 | 102553647 | T | G | 0.8875 | 0.3633 | 0.0275 | 7.96E-40 | 4.60E-05 | 174.5 |
| rs10069690 | 5 | 1279790 | T | C | 0.2581 | 0.1615 | 0.021 | 1.42E-14 | 2.99E-05 | 59.1 |
| rs10087280 | 8 | 49391836 | G | A | 0.1683 | -0.1381 | 0.0232 | 2.54E-09 | 1.31E-05 | 35.4 |
| rs10164193 | 18 | 31161426 | G | T | 0.0777 | 0.2196 | 0.0327 | 1.87E-11 | 8.53E-06 | 45.1 |
| rs1035673 | 2 | 218675533 | C | T | 0.6032 | -0.1625 | 0.0176 | 3.00E-20 | 5.39E-05 | 85.2 |
| rs1039897 | 2 | 220337196 | A | G | 0.6503 | -0.1085 | 0.0183 | 3.26E-09 | 2.11E-05 | 35.2 |
| rs1043809 | 17 | 19239432 | T | C | 0.8083 | 0.159 | 0.0223 | 9.77E-13 | 2.08E-05 | 50.8 |
| rs1044608 | 7 | 150502016 | G | C | 0.0767 | 0.2018 | 0.0339 | 2.76E-09 | 6.62E-06 | 35.4 |
| rs1044822 | 2 | 230629138 | T | C | 0.1488 | -0.1334 | 0.0243 | 4.14E-08 | 1.01E-05 | 30.1 |
| rs10490923 | 10 | 124214251 | A | G | 0.1257 | 0.1533 | 0.0262 | 5.02E-09 | 9.93E-06 | 34.2 |
| rs1049212 | 16 | 4932929 | G | A | 0.5694 | 0.1788 | 0.0175 | 1.30E-24 | 6.76E-05 | 104.4 |
| rs10493408 | 1 | 66992054 | A | C | 0.1331 | 0.1584 | 0.0255 | 5.09E-10 | 1.18E-05 | 38.6 |
| rs10500932 | 11 | 22501446 | A | G | 0.0743 | 0.2784 | 0.0333 | 5.79E-17 | 1.27E-05 | 69.9 |
| rs10759697 | 9 | 117172307 | A | G | 0.4906 | 0.1308 | 0.0173 | 3.93E-14 | 3.77E-05 | 57.2 |
| rs10776752 | 1 | 113044328 | T | G | 0.0805 | 0.4573 | 0.033 | 1.25E-43 | 3.75E-05 | 192.0 |
| rs10804330 | 2 | 227185749 | C | T | 0.4329 | -0.1331 | 0.0176 | 4.60E-14 | 3.71E-05 | 57.2 |
| rs10832586 | 11 | 16304089 | C | A | 0.2016 | 0.3083 | 0.0216 | 2.53E-46 | 8.66E-05 | 203.7 |
| rs10838702 | 11 | 47410888 | T | G | 0.3875 | 0.2375 | 0.0178 | 1.27E-40 | 0.000111546 | 178.0 |
| rs10873612 | 15 | 26105602 | T | C | 0.5961 | -0.1096 | 0.0179 | 9.51E-10 | 2.38E-05 | 37.5 |
| rs10941043 | 5 | 33194751 | G | T | 0.2906 | 0.1269 | 0.019 | 2.52E-11 | 2.43E-05 | 44.6 |
| rs10980408 | 9 | 113249071 | C | T | 0.0358 | 0.3745 | 0.0477 | 4.17E-15 | 5.62E-06 | 61.6 |
| rs11021221 | 11 | 95308854 | A | T | 0.1668 | -0.1877 | 0.0233 | 6.93E-16 | 2.38E-05 | 64.9 |
| rs11040503 | 11 | 49760350 | A | C | 0.1857 | -0.17 | 0.0236 | 5.65E-13 | 2.07E-05 | 51.9 |
| rs11070245 | 15 | 40317792 | G | T | 0.5321 | 0.1287 | 0.0174 | 1.57E-13 | 3.60E-05 | 54.7 |
| rs11108209 | 12 | 96109855 | C | T | 0.0932 | 0.1901 | 0.03 | 2.40E-10 | 8.96E-06 | 40.2 |
| rs11112548 | 12 | 105871914 | T | A | 0.0444 | -0.2742 | 0.0443 | 5.80E-10 | 4.29E-06 | 38.3 |
| rs11130602 | 3 | 57947168 | A | G | 0.4427 | 0.1467 | 0.0175 | 4.27E-17 | 4.58E-05 | 70.3 |
| rs11141731 | 9 | 89888472 | T | C | 0.228 | -0.1258 | 0.0207 | 1.31E-09 | 1.72E-05 | 36.9 |
| rs1114347 | 6 | 51834297 | G | A | 0.4823 | 0.1792 | 0.0173 | 3.32E-25 | 7.07E-05 | 107.3 |
| rs11145807 | 9 | 139520789 | G | A | 0.5942 | -0.155 | 0.0184 | 4.10E-17 | 4.52E-05 | 71.0 |
| rs11153590 | 6 | 116322381 | A | G | 0.3831 | -0.1145 | 0.0178 | 1.12E-10 | 2.58E-05 | 41.4 |
| rs11153730 | 6 | 118667522 | C | T | 0.4906 | -0.1551 | 0.0173 | 2.57E-19 | 5.30E-05 | 80.4 |
| rs11187838 | 10 | 96038686 | A | G | 0.4321 | -0.2781 | 0.0174 | 2.55E-57 | 0.000165481 | 255.4 |
| rs11190746 | 10 | 102663565 | A | G | 0.5446 | -0.11 | 0.0175 | 3.48E-10 | 2.59E-05 | 39.5 |
| rs11191580 | 10 | 104906211 | C | T | 0.0821 | -0.5071 | 0.0316 | 6.63E-58 | 5.12E-05 | 257.5 |
| rs11231693 | 11 | 63862612 | A | G | 0.0574 | 0.2223 | 0.0378 | 4.02E-09 | 4.94E-06 | 34.6 |
| rs112393817 | 2 | 9807226 | G | C | 0.2174 | -0.116 | 0.0211 | 3.80E-08 | 1.36E-05 | 30.2 |
| rs11252324 | 10 | 4124568 | T | G | 0.077 | -0.2339 | 0.0328 | 1.03E-12 | 9.54E-06 | 50.9 |
| rs1133400 | 10 | 134459388 | G | A | 0.2148 | 0.1318 | 0.0215 | 8.30E-10 | 1.67E-05 | 37.6 |
| rs114503346 | 5 | 172192350 | T | C | 0.0461 | -0.2678 | 0.0426 | 3.10E-10 | 4.59E-06 | 39.5 |
| rs114714860 | 3 | 41882905 | C | G | 0.1683 | 0.33 | 0.0236 | 1.42E-44 | 7.23E-05 | 195.5 |
| rs115447786 | 6 | 34354073 | T | C | 0.0427 | 0.2904 | 0.0455 | 1.75E-10 | 4.40E-06 | 40.7 |
| rs11556924 | 7 | 129663496 | T | C | 0.3827 | -0.181 | 0.0181 | 1.83E-23 | 6.24E-05 | 100.0 |
| rs11592107 | 10 | 122968964 | A | G | 0.3094 | 0.1203 | 0.0187 | 1.23E-10 | 2.33E-05 | 41.4 |
| rs116063464 | 12 | 109860182 | A | G | 0.0601 | 0.2017 | 0.0369 | 4.68E-08 | 4.46E-06 | 29.9 |
| rs11636952 | 15 | 75114322 | C | T | 0.6869 | -0.3997 | 0.0189 | 5.21E-99 | 0.000253928 | 447.2 |
| rs11661473 | 18 | 42177123 | A | G | 0.2683 | 0.2007 | 0.0196 | 1.54E-24 | 5.43E-05 | 104.9 |
| rs11665020 | 18 | 10879503 | C | G | 0.322 | -0.1423 | 0.0187 | 2.78E-14 | 3.34E-05 | 57.9 |
| rs11684340 | 2 | 37207251 | C | A | 0.2176 | -0.1249 | 0.021 | 2.75E-09 | 1.59E-05 | 35.4 |
| rs11692619 | 2 | 205084439 | T | C | 0.3607 | -0.1281 | 0.0184 | 3.31E-12 | 2.95E-05 | 48.5 |
| rs11721984 | 4 | 38343935 | T | C | 0.4532 | -0.1409 | 0.0177 | 1.89E-15 | 4.15E-05 | 63.4 |
| rs117464403 | 10 | 107158054 | A | G | 0.0184 | 0.4537 | 0.0682 | 2.95E-11 | 2.11E-06 | 44.3 |
| rs11778153 | 8 | 64503942 | C | T | 0.3569 | -0.1192 | 0.0182 | 5.84E-11 | 2.60E-05 | 42.9 |
| rs1178979 | 7 | 72856430 | C | T | 0.1953 | -0.1504 | 0.0221 | 9.96E-12 | 1.92E-05 | 46.3 |
| rs11859505 | 16 | 74195719 | G | A | 0.5805 | 0.1037 | 0.0181 | 9.76E-09 | 2.11E-05 | 32.8 |
| rs11923343 | 3 | 85668570 | G | A | 0.6396 | 0.1138 | 0.0181 | 3.10E-10 | 2.41E-05 | 39.5 |
| rs11923667 | 3 | 101268080 | A | T | 0.4071 | 0.1175 | 0.0177 | 3.10E-11 | 2.81E-05 | 44.1 |
| rs11945489 | 4 | 56463775 | T | C | 0.2909 | -0.1392 | 0.0192 | 3.99E-13 | 2.86E-05 | 52.6 |
| rs11960210 | 5 | 157817634 | C | T | 0.3751 | -0.2474 | 0.018 | 3.36E-43 | 0.000116896 | 188.9 |
| rs11961593 | 6 | 166164137 | T | C | 0.0685 | -0.3158 | 0.0349 | 1.49E-19 | 1.38E-05 | 81.9 |
| rs12088448 | 1 | 218546170 | C | A | 0.356 | 0.1544 | 0.0182 | 2.53E-17 | 4.36E-05 | 72.0 |
| rs12148044 | 14 | 69214219 | A | G | 0.1734 | 0.1371 | 0.023 | 2.66E-09 | 1.34E-05 | 35.5 |
| rs12149254 | 16 | 71458851 | A | G | 0.1697 | -0.1324 | 0.0232 | 1.14E-08 | 1.21E-05 | 32.6 |
| rs12152463 | 3 | 122100447 | T | C | 0.4251 | 0.1006 | 0.0174 | 8.01E-09 | 2.16E-05 | 33.4 |
| rs1215469 | 13 | 80707408 | C | A | 0.7705 | 0.1383 | 0.0211 | 5.23E-11 | 2.01E-05 | 43.0 |
| rs12216886 | 9 | 2493751 | G | T | 0.1923 | -0.1292 | 0.0221 | 4.76E-09 | 1.40E-05 | 34.2 |
| rs12228145 | 12 | 53510852 | T | C | 0.1518 | -0.1336 | 0.0244 | 4.64E-08 | 1.02E-05 | 30.0 |
| rs12247028 | 10 | 75410052 | A | G | 0.6322 | -0.1396 | 0.0188 | 1.18E-13 | 3.38E-05 | 55.1 |
| rs12258967 | 10 | 18727959 | G | C | 0.2958 | -0.354 | 0.0193 | 3.27E-75 | 0.000185002 | 336.4 |
| rs12337056 | 9 | 628670 | T | C | 0.1761 | 0.1364 | 0.0228 | 2.18E-09 | 1.37E-05 | 35.8 |
| rs12363520 | 11 | 10692172 | A | T | 0.229 | 0.1672 | 0.0213 | 4.24E-15 | 2.87E-05 | 61.6 |
| rs12405515 | 1 | 172357441 | T | G | 0.5702 | -0.1698 | 0.0174 | 1.92E-22 | 6.16E-05 | 95.2 |
| rs1243876 | 9 | 35693104 | T | C | 0.7012 | -0.1063 | 0.019 | 2.14E-08 | 1.73E-05 | 31.3 |
| rs12503341 | 4 | 106925311 | A | G | 0.0394 | -0.2993 | 0.0462 | 9.43E-11 | 4.19E-06 | 42.0 |
| rs12509595 | 4 | 81182554 | C | T | 0.2924 | 0.4972 | 0.0192 | 1.58E-148 | 0.000366281 | 670.6 |
| rs12515541 | 5 | 57095011 | T | G | 0.6072 | 0.1156 | 0.0177 | 6.23E-11 | 2.69E-05 | 42.7 |
| rs12574332 | 11 | 122521123 | T | C | 0.1227 | 0.2072 | 0.0266 | 6.14E-15 | 1.72E-05 | 60.7 |
| rs12596630 | 16 | 2065666 | T | C | 0.0905 | 0.2606 | 0.0314 | 1.03E-16 | 1.50E-05 | 68.9 |
| rs12601936 | 17 | 7172609 | G | A | 0.6107 | 0.1429 | 0.0178 | 1.07E-15 | 4.05E-05 | 64.5 |
| rs12609484 | 19 | 4970593 | T | G | 0.3163 | -0.1398 | 0.0188 | 1.16E-13 | 3.16E-05 | 55.3 |
| rs12627514 | 21 | 44759440 | G | C | 0.2897 | 0.2164 | 0.0196 | 1.99E-28 | 6.62E-05 | 121.9 |
| rs1263671 | 2 | 207996447 | C | T | 0.1632 | 0.1394 | 0.0238 | 4.69E-09 | 1.24E-05 | 34.3 |
| rs1265157 | 6 | 31142265 | G | C | 0.3521 | -0.1444 | 0.0187 | 1.18E-14 | 3.59E-05 | 59.6 |
| rs12656497 | 5 | 32831939 | C | T | 0.5968 | 0.3063 | 0.0176 | 1.47E-67 | 0.000192401 | 302.9 |
| rs1265842 | 10 | 28924901 | C | T | 0.5166 | -0.1113 | 0.0174 | 1.70E-10 | 2.70E-05 | 40.9 |
| rs12693302 | 2 | 183211443 | A | G | 0.6518 | -0.2378 | 0.0181 | 2.15E-39 | 0.000103419 | 172.6 |
| rs12699415 | 7 | 1909479 | G | A | 0.5873 | -0.1236 | 0.0177 | 3.30E-12 | 3.12E-05 | 48.8 |
| rs12728150 | 1 | 27268737 | G | A | 0.081 | 0.2045 | 0.0318 | 1.28E-10 | 8.13E-06 | 41.4 |
| rs1275985 | 2 | 26911745 | T | C | 0.6133 | -0.2943 | 0.0177 | 2.77E-62 | 0.000173089 | 276.5 |
| rs12790943 | 11 | 120058623 | T | C | 0.4213 | -0.1002 | 0.0175 | 1.14E-08 | 2.11E-05 | 32.8 |
| rs12866098 | 13 | 73119617 | A | G | 0.3423 | 0.1033 | 0.0186 | 2.73E-08 | 1.83E-05 | 30.8 |
| rs12906962 | 15 | 95312071 | C | T | 0.3233 | 0.2378 | 0.0188 | 8.73E-37 | 9.24E-05 | 160.0 |
| rs12919839 | 16 | 56859216 | T | C | 0.2841 | -0.1098 | 0.0192 | 1.04E-08 | 1.76E-05 | 32.7 |
| rs12929303 | 16 | 81602264 | A | G | 0.5325 | 0.1572 | 0.0174 | 1.58E-19 | 5.36E-05 | 81.6 |
| rs12978472 | 19 | 7257990 | G | C | 0.1247 | -0.4779 | 0.0281 | 8.46E-65 | 8.33E-05 | 289.2 |
| rs12990959 | 2 | 148572160 | C | T | 0.3125 | 0.1271 | 0.0187 | 1.11E-11 | 2.62E-05 | 46.2 |
| rs13004222 | 2 | 219560492 | G | C | 0.051 | -0.2944 | 0.0393 | 7.11E-14 | 7.17E-06 | 56.1 |
| rs13021015 | 2 | 127183453 | A | C | 0.1596 | 0.1516 | 0.0239 | 2.29E-10 | 1.42E-05 | 40.2 |
| rs13107325 | 4 | 103188709 | T | C | 0.0742 | -0.6747 | 0.0339 | 3.72E-88 | 7.18E-05 | 396.1 |
| rs13118687 | 4 | 111406496 | A | G | 0.4702 | -0.1496 | 0.0175 | 1.37E-17 | 4.81E-05 | 73.1 |
| rs13124515 | 4 | 145403713 | C | T | 0.6869 | 0.1052 | 0.0187 | 1.98E-08 | 1.80E-05 | 31.6 |
| rs13139571 | 4 | 156645513 | A | C | 0.2366 | -0.2408 | 0.0203 | 2.29E-32 | 6.71E-05 | 140.7 |
| rs13152154 | 4 | 77417756 | T | C | 0.7293 | -0.1186 | 0.0195 | 1.23E-09 | 1.93E-05 | 37.0 |
| rs13215166 | 6 | 127164360 | G | A | 0.4415 | 0.3094 | 0.0174 | 1.79E-70 | 0.000205819 | 316.2 |
| rs1322639 | 6 | 169587103 | A | G | 0.7766 | -0.1584 | 0.0209 | 3.87E-14 | 2.63E-05 | 57.4 |
| rs13237249 | 7 | 131151783 | T | C | 0.398 | 0.1366 | 0.0177 | 1.02E-14 | 3.77E-05 | 59.6 |
| rs1327235 | 20 | 10969030 | G | A | 0.4714 | 0.3018 | 0.0173 | 4.76E-68 | 0.000200195 | 304.3 |
| rs1332812 | 9 | 9350986 | A | T | 0.6469 | -0.1145 | 0.0181 | 2.71E-10 | 2.41E-05 | 40.0 |
| rs13355146 | 5 | 92023661 | T | C | 0.3832 | 0.1224 | 0.0178 | 6.39E-12 | 2.95E-05 | 47.3 |
| rs13358657 | 5 | 157938070 | G | A | 0.1332 | 0.224 | 0.0255 | 1.70E-18 | 2.35E-05 | 77.2 |
| rs135023 | 22 | 32442776 | G | A | 0.5784 | 0.1036 | 0.0175 | 3.53E-09 | 2.26E-05 | 35.0 |
| rs1373780 | 2 | 19501029 | C | G | 0.1845 | 0.1246 | 0.0224 | 2.58E-08 | 1.23E-05 | 30.9 |
| rs138420351 | 17 | 7700063 | T | C | 0.016 | 0.5568 | 0.0854 | 7.11E-11 | 1.77E-06 | 42.5 |
| rs142449193 | 8 | 102750597 | T | C | 0.046 | -0.2573 | 0.0426 | 1.51E-09 | 4.23E-06 | 36.5 |
| rs1425486 | 4 | 157683685 | T | C | 0.3207 | -0.1331 | 0.0187 | 1.11E-12 | 2.91E-05 | 50.7 |
| rs143549627 | 12 | 57797043 | A | G | 0.0354 | -0.3179 | 0.0498 | 1.73E-10 | 3.67E-06 | 40.7 |
| rs1436138 | 17 | 75316880 | G | A | 0.3633 | -0.1991 | 0.0182 | 7.33E-28 | 7.31E-05 | 119.7 |
| rs1446468 | 2 | 164963486 | C | T | 0.546 | 0.253 | 0.0174 | 1.21E-47 | 0.00013835 | 211.4 |
| rs1449596 | 7 | 96395096 | G | C | 0.6455 | 0.1085 | 0.0181 | 1.92E-09 | 2.17E-05 | 35.9 |
| rs145422110 | 11 | 1830431 | T | C | 0.0162 | 0.5356 | 0.0745 | 6.41E-13 | 2.17E-06 | 51.7 |
| rs147501096 | 3 | 186180253 | C | G | 0.072 | -0.1955 | 0.0341 | 9.94E-09 | 5.80E-06 | 32.9 |
| rs148401029 | 8 | 81386066 | A | C | 0.0352 | -0.3122 | 0.0486 | 1.32E-10 | 3.70E-06 | 41.3 |
| rs1493132 | 4 | 108861082 | C | T | 0.3406 | 0.1191 | 0.0182 | 6.37E-11 | 2.54E-05 | 42.8 |
| rs1502358 | 1 | 217324932 | A | G | 0.6813 | -0.1127 | 0.0185 | 1.13E-09 | 2.13E-05 | 37.1 |
| rs150816167 | 1 | 179571862 | C | T | 0.0451 | 0.2873 | 0.0446 | 1.17E-10 | 4.72E-06 | 41.5 |
| rs1518460 | 2 | 181933689 | G | A | 0.2918 | -0.1342 | 0.0189 | 1.26E-12 | 2.75E-05 | 50.4 |
| rs1582931 | 5 | 122657199 | A | G | 0.4748 | 0.2161 | 0.0175 | 4.51E-35 | 0.000100383 | 152.5 |
| rs1623474 | 10 | 18471794 | T | C | 0.33 | 0.2234 | 0.0184 | 6.24E-34 | 8.60E-05 | 147.4 |
| rs1669907 | 12 | 42777933 | G | T | 0.6968 | -0.1158 | 0.0191 | 1.36E-09 | 2.05E-05 | 36.8 |
| rs16853198 | 3 | 168840179 | G | A | 0.0762 | -0.3386 | 0.0327 | 4.44E-25 | 1.99E-05 | 107.2 |
| rs1687295 | 3 | 14889756 | C | T | 0.7296 | -0.2061 | 0.0194 | 2.99E-26 | 5.88E-05 | 112.9 |
| rs16875357 | 6 | 85652904 | G | T | 0.2431 | 0.1205 | 0.0203 | 2.70E-09 | 1.71E-05 | 35.2 |
| rs16896276 | 4 | 18015156 | A | T | 0.2625 | -0.1309 | 0.0198 | 3.82E-11 | 2.23E-05 | 43.7 |
| rs1693560 | 8 | 101680292 | G | A | 0.4603 | -0.1525 | 0.0175 | 3.41E-18 | 4.98E-05 | 75.9 |
| rs17321041 | 8 | 26445194 | T | C | 0.0633 | 0.2313 | 0.0363 | 1.78E-10 | 6.36E-06 | 40.6 |
| rs1732664 | 12 | 79747487 | C | T | 0.6783 | 0.1083 | 0.0194 | 2.58E-08 | 1.80E-05 | 31.2 |
| rs17396055 | 1 | 94730954 | A | G | 0.3324 | -0.115 | 0.0184 | 4.13E-10 | 2.29E-05 | 39.1 |
| rs17432462 | 7 | 18548613 | C | T | 0.3766 | 0.1036 | 0.0179 | 7.31E-09 | 2.08E-05 | 33.5 |
| rs17454517 | 7 | 50915776 | G | A | 0.5064 | -0.1216 | 0.0174 | 2.65E-12 | 3.22E-05 | 48.8 |
| rs17677603 | 5 | 127857493 | G | A | 0.3837 | 0.2 | 0.0178 | 3.90E-29 | 7.88E-05 | 126.2 |
| rs17678552 | 15 | 42066190 | C | T | 0.3439 | 0.1649 | 0.0182 | 1.33E-19 | 4.89E-05 | 82.1 |
| rs17832905 | 8 | 26038759 | A | C | 0.0717 | 0.1923 | 0.0346 | 2.80E-08 | 5.43E-06 | 30.9 |
| rs17880989 | 14 | 23313633 | A | G | 0.0259 | 0.4014 | 0.0591 | 1.11E-11 | 3.07E-06 | 46.1 |
| rs1790123 | 12 | 123659542 | T | C | 0.8032 | 0.1991 | 0.0218 | 6.87E-20 | 3.48E-05 | 83.4 |
| rs1799945 | 6 | 26091179 | G | C | 0.1497 | 0.3888 | 0.0244 | 3.88E-57 | 8.53E-05 | 253.9 |
| rs1819663 | 1 | 154025891 | G | A | 0.4929 | -0.1147 | 0.0174 | 4.62E-11 | 2.87E-05 | 43.5 |
| rs1867624 | 17 | 62387091 | T | C | 0.6147 | 0.1412 | 0.0178 | 2.08E-15 | 3.93E-05 | 62.9 |
| rs1871190 | 5 | 97953719 | T | G | 0.3344 | 0.1078 | 0.0186 | 6.63E-09 | 1.97E-05 | 33.6 |
| rs1876490 | 2 | 73052351 | A | G | 0.7167 | 0.1364 | 0.0192 | 1.16E-12 | 2.71E-05 | 50.5 |
| rs1882961 | 21 | 16556367 | T | C | 0.3088 | 0.1272 | 0.0188 | 1.40E-11 | 2.58E-05 | 45.8 |
| rs1889785 | 1 | 16348729 | A | G | 0.4551 | 0.1255 | 0.0174 | 5.61E-13 | 3.41E-05 | 52.0 |
| rs1903752 | 18 | 7129327 | T | C | 0.5386 | -0.0987 | 0.0178 | 3.20E-08 | 2.02E-05 | 30.7 |
| rs1906672 | 8 | 38130025 | A | G | 0.2324 | 0.1402 | 0.0205 | 8.47E-12 | 2.20E-05 | 46.8 |
| rs1948151 | 12 | 26459071 | A | G | 0.2777 | -0.1355 | 0.0193 | 2.12E-12 | 2.61E-05 | 49.3 |
| rs1950500 | 14 | 24830850 | C | T | 0.7081 | -0.1396 | 0.019 | 2.20E-13 | 2.95E-05 | 54.0 |
| rs1981405 | 11 | 77976208 | T | C | 0.1248 | -0.1519 | 0.0266 | 1.16E-08 | 9.40E-06 | 32.6 |
| rs1984195 | 6 | 79657391 | A | G | 0.4883 | 0.1736 | 0.0173 | 1.43E-23 | 6.64E-05 | 100.7 |
| rs2062011 | 18 | 60876114 | A | T | 0.2491 | -0.1138 | 0.0209 | 4.80E-08 | 1.46E-05 | 29.6 |
| rs2098702 | 5 | 57761287 | A | G | 0.3252 | 0.1143 | 0.0185 | 6.50E-10 | 2.21E-05 | 38.2 |
| rs2133386 | 9 | 128173838 | A | C | 0.4327 | -0.1322 | 0.0176 | 5.21E-14 | 3.66E-05 | 56.4 |
| rs2146315 | 1 | 42050366 | T | C | 0.2318 | -0.1197 | 0.0205 | 5.03E-09 | 1.60E-05 | 34.1 |
| rs2160236 | 2 | 40557276 | C | G | 0.3792 | -0.1421 | 0.0181 | 4.31E-15 | 3.83E-05 | 61.6 |
| rs2169137 | 1 | 204497913 | C | G | 0.7287 | 0.1588 | 0.0194 | 3.17E-16 | 3.50E-05 | 67.0 |
| rs2191046 | 7 | 107834075 | G | T | 0.2646 | -0.1184 | 0.0197 | 1.78E-09 | 1.86E-05 | 36.1 |
| rs2236295 | 10 | 64564892 | T | G | 0.3992 | -0.207 | 0.0177 | 1.42E-31 | 8.66E-05 | 136.8 |
| rs2239268 | 14 | 72469591 | A | G | 0.7005 | 0.1097 | 0.019 | 7.40E-09 | 1.85E-05 | 33.3 |
| rs2239917 | 17 | 43165887 | C | T | 0.5748 | -0.1731 | 0.0176 | 9.69E-23 | 6.24E-05 | 96.7 |
| rs2271139 | 12 | 124839540 | A | C | 0.286 | -0.1247 | 0.0192 | 8.23E-11 | 2.27E-05 | 42.2 |
| rs227426 | 14 | 70456664 | T | G | 0.5619 | 0.1119 | 0.0175 | 1.75E-10 | 2.66E-05 | 40.9 |
| rs2291516 | 19 | 11508177 | A | G | 0.1026 | 0.2422 | 0.0292 | 9.88E-17 | 1.67E-05 | 68.8 |
| rs2307111 | 5 | 75003678 | C | T | 0.3966 | 0.1742 | 0.0178 | 1.62E-22 | 6.05E-05 | 95.8 |
| rs234622 | 20 | 57489636 | G | C | 0.3356 | -0.114 | 0.0185 | 6.62E-10 | 2.24E-05 | 38.0 |
| rs2384061 | 2 | 25135620 | A | G | 0.4221 | -0.1741 | 0.0175 | 2.25E-23 | 6.37E-05 | 99.0 |
| rs2397060 | 6 | 51611470 | C | T | 0.1405 | 0.161 | 0.0251 | 1.46E-10 | 1.31E-05 | 41.1 |
| rs2421200 | 2 | 61711815 | T | G | 0.4882 | -0.1097 | 0.0173 | 2.59E-10 | 2.65E-05 | 40.2 |
| rs2444769 | 2 | 158494100 | A | C | 0.7949 | 0.158 | 0.0219 | 4.85E-13 | 2.24E-05 | 52.1 |
| rs2469141 | 15 | 66967398 | C | T | 0.1628 | -0.1351 | 0.0238 | 1.39E-08 | 1.16E-05 | 32.2 |
| rs2484294 | 10 | 115792062 | A | G | 0.7327 | 0.3165 | 0.0196 | 1.17E-58 | 0.000134819 | 260.8 |
| rs2487926 | 10 | 30300787 | G | A | 0.4295 | -0.0972 | 0.0176 | 3.31E-08 | 1.97E-05 | 30.5 |
| rs2493136 | 1 | 230851536 | T | C | 0.4075 | 0.2345 | 0.0176 | 1.92E-40 | 0.000113153 | 177.5 |
| rs2493296 | 1 | 3327032 | T | C | 0.1419 | 0.2496 | 0.0254 | 7.45E-23 | 3.10E-05 | 96.6 |
| rs2515424 | 8 | 6372965 | T | C | 0.4336 | 0.1278 | 0.0174 | 2.18E-13 | 3.50E-05 | 53.9 |
| rs2548459 | 19 | 49209339 | C | T | 0.5195 | 0.132 | 0.0176 | 5.95E-14 | 3.71E-05 | 56.3 |
| rs2569882 | 6 | 1620147 | C | T | 0.4342 | -0.1199 | 0.0182 | 4.28E-11 | 2.81E-05 | 43.4 |
| rs2586970 | 2 | 55829967 | G | A | 0.5639 | 0.1493 | 0.0175 | 1.56E-17 | 4.73E-05 | 72.8 |
| rs2589218 | 15 | 96785017 | C | T | 0.2698 | 0.1207 | 0.0196 | 6.90E-10 | 1.97E-05 | 37.9 |
| rs2598 | 20 | 47241618 | G | A | 0.4674 | -0.1387 | 0.0175 | 1.94E-15 | 4.13E-05 | 62.8 |
| rs2627313 | 15 | 81006712 | T | C | 0.4457 | 0.151 | 0.0175 | 5.85E-18 | 4.86E-05 | 74.5 |
| rs2643826 | 3 | 27562988 | T | C | 0.4508 | 0.1857 | 0.0175 | 2.83E-26 | 7.36E-05 | 112.6 |
| rs2681485 | 12 | 90025622 | A | G | 0.5976 | 0.2945 | 0.0176 | 1.31E-62 | 0.000177747 | 280.0 |
| rs2744133 | 6 | 22392260 | G | A | 0.2749 | -0.1435 | 0.0193 | 1.17E-13 | 2.91E-05 | 55.3 |
| rs28377357 | 2 | 112769721 | A | G | 0.2938 | -0.1243 | 0.019 | 6.03E-11 | 2.34E-05 | 42.8 |
| rs28429256 | 15 | 66931617 | A | G | 0.3344 | 0.1636 | 0.0188 | 2.83E-18 | 4.45E-05 | 75.7 |
| rs28544928 | 16 | 69329268 | G | T | 0.2535 | -0.1543 | 0.0199 | 9.13E-15 | 3.00E-05 | 60.1 |
| rs28661492 | 17 | 30609932 | T | C | 0.2022 | -0.1359 | 0.0222 | 9.56E-10 | 1.60E-05 | 37.5 |
| rs28667801 | 4 | 26785356 | T | A | 0.407 | 0.1622 | 0.018 | 1.90E-19 | 5.17E-05 | 81.2 |
| rs28675079 | 3 | 111500002 | A | G | 0.1867 | -0.1444 | 0.0222 | 8.34E-11 | 1.70E-05 | 42.3 |
| rs2906152 | 7 | 2523003 | A | G | 0.6304 | -0.1873 | 0.0181 | 5.55E-25 | 6.59E-05 | 107.1 |
| rs2921604 | 5 | 14867948 | C | T | 0.4633 | 0.096 | 0.0176 | 4.46E-08 | 1.95E-05 | 29.8 |
| rs2925345 | 15 | 41311799 | C | T | 0.5324 | -0.189 | 0.0174 | 1.60E-27 | 7.75E-05 | 118.0 |
| rs2957468 | 8 | 106325360 | G | A | 0.6646 | -0.1377 | 0.0185 | 8.43E-14 | 3.26E-05 | 55.4 |
| rs311564 | 2 | 86293498 | A | G | 0.3461 | -0.133 | 0.0183 | 4.23E-13 | 3.16E-05 | 52.8 |
| rs3117736 | 5 | 157462999 | T | C | 0.2661 | 0.2374 | 0.0196 | 9.71E-34 | 7.56E-05 | 146.7 |
| rs318712 | 19 | 11478847 | C | T | 0.0759 | 0.2389 | 0.0334 | 9.17E-13 | 9.47E-06 | 51.2 |
| rs34130368 | 10 | 48411796 | T | G | 0.1172 | -0.2027 | 0.0284 | 8.77E-13 | 1.39E-05 | 50.9 |
| rs342977 | 7 | 35459888 | A | G | 0.7715 | -0.1577 | 0.0205 | 1.67E-14 | 2.75E-05 | 59.2 |
| rs34413141 | 18 | 777282 | A | T | 0.1821 | -0.1808 | 0.0227 | 1.49E-15 | 2.49E-05 | 63.4 |
| rs34487963 | 21 | 44838330 | A | C | 0.0185 | -0.5734 | 0.0712 | 8.18E-16 | 3.11E-06 | 64.9 |
| rs34517439 | 1 | 78450517 | A | C | 0.1199 | -0.2514 | 0.0279 | 2.02E-19 | 2.26E-05 | 81.2 |
| rs34587839 | 20 | 32300671 | A | G | 0.1535 | -0.1669 | 0.0244 | 8.22E-12 | 1.60E-05 | 46.8 |
| rs34645159 | 1 | 1724366 | A | G | 0.5013 | -0.133 | 0.0174 | 2.07E-14 | 3.86E-05 | 58.4 |
| rs347585 | 3 | 11286220 | T | C | 0.7014 | 0.1506 | 0.0189 | 1.57E-15 | 3.51E-05 | 63.5 |
| rs35091929 | 8 | 10693492 | C | T | 0.6032 | -0.1828 | 0.0177 | 6.46E-25 | 6.74E-05 | 106.7 |
| rs35213536 | 20 | 62694319 | T | G | 0.2467 | 0.2044 | 0.0205 | 2.54E-23 | 4.88E-05 | 99.4 |
| rs35261542 | 6 | 20675792 | A | C | 0.2679 | 0.1196 | 0.0195 | 9.29E-10 | 1.95E-05 | 37.6 |
| rs35413927 | 14 | 53420358 | G | A | 0.3049 | 0.1274 | 0.0189 | 1.77E-11 | 2.54E-05 | 45.4 |
| rs35443 | 12 | 115552878 | C | G | 0.3858 | -0.2661 | 0.0178 | 1.20E-50 | 0.000139801 | 223.5 |
| rs35506078 | 10 | 65210552 | C | T | 0.3366 | 0.1348 | 0.0183 | 1.54E-13 | 3.20E-05 | 54.3 |
| rs36117336 | 3 | 153732232 | C | T | 0.2562 | 0.147 | 0.0198 | 1.10E-13 | 2.77E-05 | 55.1 |
| rs3735533 | 7 | 27245893 | C | T | 0.9258 | 0.487 | 0.0331 | 6.32E-49 | 3.93E-05 | 216.5 |
| rs3743111 | 15 | 71587373 | A | G | 0.613 | 0.1517 | 0.0178 | 1.62E-17 | 4.55E-05 | 72.6 |
| rs3743369 | 15 | 92707569 | A | G | 0.6278 | 0.104 | 0.0179 | 6.82E-09 | 2.08E-05 | 33.8 |
| rs3761077 | 19 | 19325963 | T | G | 0.1106 | 0.1726 | 0.0283 | 1.04E-09 | 9.66E-06 | 37.2 |
| rs3772219 | 3 | 56771251 | C | A | 0.3193 | -0.1754 | 0.0185 | 2.94E-21 | 5.16E-05 | 89.9 |
| rs3774702 | 3 | 63856870 | A | G | 0.1768 | 0.147 | 0.0228 | 1.18E-10 | 1.60E-05 | 41.6 |
| rs3776299 | 5 | 142507651 | A | G | 0.4559 | 0.1266 | 0.0175 | 5.06E-13 | 3.43E-05 | 52.3 |
| rs3785837 | 17 | 59468942 | A | G | 0.7635 | 0.1453 | 0.0213 | 9.57E-12 | 2.22E-05 | 46.5 |
| rs378825 | 11 | 9766932 | G | A | 0.5791 | 0.2143 | 0.0175 | 1.90E-34 | 9.65E-05 | 150.0 |
| rs3798293 | 6 | 97033370 | G | A | 0.2165 | 0.1328 | 0.021 | 2.70E-10 | 1.79E-05 | 40.0 |
| rs3802230 | 8 | 143992864 | A | C | 0.5446 | -0.1605 | 0.0174 | 2.75E-20 | 5.57E-05 | 85.1 |
| rs3807101 | 7 | 80393418 | T | C | 0.123 | -0.1743 | 0.0265 | 4.57E-11 | 1.23E-05 | 43.3 |
| rs3861113 | 13 | 72364382 | A | C | 0.0825 | 0.2126 | 0.0322 | 3.95E-11 | 8.71E-06 | 43.6 |
| rs3864004 | 3 | 41240177 | A | G | 0.4685 | 0.1004 | 0.0173 | 6.28E-09 | 2.21E-05 | 33.7 |
| rs3916033 | 17 | 44889703 | T | C | 0.5565 | -0.1233 | 0.0185 | 2.41E-11 | 2.89E-05 | 44.4 |
| rs3918226 | 7 | 150690176 | T | C | 0.0813 | 0.6117 | 0.0329 | 5.31E-77 | 6.82E-05 | 345.7 |
| rs3943093 | 1 | 243458502 | T | C | 0.3234 | 0.2477 | 0.0184 | 3.94E-41 | 0.000104683 | 181.2 |
| rs4074812 | 8 | 141883529 | A | G | 0.5535 | -0.1336 | 0.0175 | 2.07E-14 | 3.80E-05 | 58.3 |
| rs4077158 | 3 | 133942941 | C | T | 0.5286 | 0.1832 | 0.0173 | 3.09E-26 | 7.38E-05 | 112.1 |
| rs4102481 | 18 | 51774715 | G | T | 0.3049 | 0.1248 | 0.019 | 4.87E-11 | 2.41E-05 | 43.1 |
| rs4141663 | 3 | 124551967 | T | C | 0.4216 | -0.1496 | 0.0175 | 1.41E-17 | 4.70E-05 | 73.1 |
| rs4143175 | 12 | 67782397 | C | T | 0.7589 | -0.1139 | 0.0203 | 2.11E-08 | 1.52E-05 | 31.5 |
| rs4244200 | 3 | 196226059 | C | G | 0.2799 | -0.1215 | 0.0193 | 3.23E-10 | 2.11E-05 | 39.6 |
| rs4284362 | 10 | 45377839 | A | C | 0.7181 | -0.1586 | 0.0194 | 3.24E-16 | 3.57E-05 | 66.8 |
| rs4295 | 17 | 61556298 | G | C | 0.6202 | -0.1802 | 0.018 | 1.71E-23 | 6.23E-05 | 100.2 |
| rs4306343 | 12 | 20190630 | T | A | 0.7212 | 0.317 | 0.0193 | 8.22E-61 | 0.0001432 | 269.8 |
| rs4362428 | 17 | 2090341 | A | C | 0.4087 | -0.1127 | 0.0176 | 1.45E-10 | 2.62E-05 | 41.0 |
| rs440454 | 6 | 31927342 | G | A | 0.684 | 0.2602 | 0.0192 | 7.52E-42 | 0.000104796 | 183.7 |
| rs4424827 | 14 | 35110857 | T | C | 0.5669 | -0.0981 | 0.0175 | 2.11E-08 | 2.04E-05 | 31.4 |
| rs4507125 | 2 | 239864732 | C | A | 0.2136 | 0.1244 | 0.0211 | 3.60E-09 | 1.54E-05 | 34.8 |
| rs4507656 | 7 | 22156538 | G | C | 0.3066 | 0.1487 | 0.0199 | 8.69E-14 | 3.13E-05 | 55.8 |
| rs45474499 | 16 | 66914492 | T | C | 0.0473 | 0.3562 | 0.0415 | 8.50E-18 | 8.76E-06 | 73.7 |
| rs4556017 | 7 | 100632790 | T | C | 0.8524 | -0.1601 | 0.0247 | 9.66E-11 | 1.40E-05 | 42.0 |
| rs4615669 | 9 | 21818674 | G | A | 0.4403 | 0.114 | 0.0174 | 6.10E-11 | 2.79E-05 | 42.9 |
| rs4645335 | 5 | 3704761 | G | A | 0.664 | -0.1142 | 0.0185 | 7.04E-10 | 2.24E-05 | 38.1 |
| rs4651224 | 1 | 184585182 | T | C | 0.4469 | 0.1102 | 0.0175 | 3.39E-10 | 2.59E-05 | 39.7 |
| rs4673253 | 2 | 204120214 | G | C | 0.2655 | 0.1177 | 0.0197 | 2.44E-09 | 1.84E-05 | 35.7 |
| rs4675682 | 2 | 208402750 | C | T | 0.4622 | 0.1409 | 0.0173 | 4.49E-16 | 4.35E-05 | 66.3 |
| rs4704514 | 5 | 77820081 | T | C | 0.2833 | 0.1087 | 0.0193 | 1.71E-08 | 1.70E-05 | 31.7 |
| rs4722548 | 7 | 25961519 | C | T | 0.3996 | 0.1346 | 0.0176 | 1.99E-14 | 3.70E-05 | 58.5 |
| rs4726006 | 7 | 150878803 | A | G | 0.2548 | 0.1339 | 0.02 | 2.39E-11 | 2.25E-05 | 44.8 |
| rs4739832 | 8 | 82812019 | C | A | 0.4167 | -0.1323 | 0.0176 | 5.81E-14 | 3.63E-05 | 56.5 |
| rs4743021 | 9 | 109414561 | C | T | 0.3147 | 0.108 | 0.0194 | 2.41E-08 | 1.76E-05 | 31.0 |
| rs4756782 | 11 | 14254606 | A | C | 0.165 | 0.1551 | 0.0234 | 3.52E-11 | 1.60E-05 | 43.9 |
| rs4814837 | 20 | 19241680 | T | C | 0.3424 | -0.1003 | 0.0184 | 4.62E-08 | 1.77E-05 | 29.7 |
| rs4873492 | 8 | 51947549 | T | C | 0.1725 | 0.1401 | 0.0231 | 1.28E-09 | 1.39E-05 | 36.8 |
| rs488834 | 1 | 10767902 | T | C | 0.7641 | -0.1931 | 0.0208 | 1.94E-20 | 4.10E-05 | 86.2 |
| rs4891258 | 18 | 72995537 | G | A | 0.3174 | 0.1159 | 0.0187 | 5.72E-10 | 2.20E-05 | 38.4 |
| rs4903064 | 14 | 73279420 | C | T | 0.2355 | -0.1543 | 0.0206 | 7.84E-14 | 2.67E-05 | 56.1 |
| rs4909314 | 8 | 135623798 | A | T | 0.3948 | 0.1339 | 0.0177 | 3.41E-14 | 3.61E-05 | 57.2 |
| rs4912840 | 5 | 141662480 | G | A | 0.8453 | 0.1485 | 0.0245 | 1.25E-09 | 1.27E-05 | 36.7 |
| rs4918065 | 10 | 105617578 | C | T | 0.2504 | -0.1327 | 0.0201 | 3.76E-11 | 2.16E-05 | 43.6 |
| rs4926499 | 1 | 249155909 | C | G | 0.826 | 0.1694 | 0.0248 | 9.37E-12 | 1.77E-05 | 46.7 |
| rs4926901 | 1 | 48025824 | A | G | 0.3548 | 0.0984 | 0.018 | 4.82E-08 | 1.81E-05 | 29.9 |
| rs4926923 | 1 | 48109225 | C | T | 0.0883 | -0.1918 | 0.0308 | 4.75E-10 | 8.24E-06 | 38.8 |
| rs4930295 | 11 | 65390554 | G | C | 0.2216 | -0.2418 | 0.0209 | 5.54E-31 | 6.10E-05 | 133.9 |
| rs4932373 | 15 | 91429287 | C | A | 0.3257 | 0.3664 | 0.0189 | 7.71E-84 | 0.000217895 | 375.8 |
| rs4936099 | 11 | 130280725 | A | C | 0.5989 | 0.1745 | 0.0178 | 1.16E-22 | 6.09E-05 | 96.1 |
| rs4952668 | 2 | 43386568 | A | G | 0.6237 | -0.192 | 0.018 | 1.13E-26 | 7.05E-05 | 113.8 |
| rs4993969 | 16 | 75411521 | T | A | 0.5932 | 0.1265 | 0.0176 | 6.62E-13 | 3.29E-05 | 51.7 |
| rs5012479 | 6 | 109630096 | G | T | 0.5205 | -0.0956 | 0.0174 | 3.66E-08 | 1.99E-05 | 30.2 |
| rs504217 | 11 | 72006086 | T | C | 0.0736 | 0.2745 | 0.0335 | 2.51E-16 | 1.21E-05 | 67.1 |
| rs504691 | 6 | 72206620 | A | C | 0.4002 | -0.1177 | 0.0177 | 3.14E-11 | 2.80E-05 | 44.2 |
| rs507666 | 9 | 136149399 | A | G | 0.1872 | -0.2854 | 0.0223 | 2.27E-37 | 6.58E-05 | 163.8 |
| rs509067 | 6 | 117462040 | C | T | 0.5863 | 0.1436 | 0.0175 | 2.65E-16 | 4.31E-05 | 67.3 |
| rs520592 | 12 | 69939355 | G | T | 0.1413 | 0.1727 | 0.0248 | 3.51E-12 | 1.55E-05 | 48.5 |
| rs55684003 | 13 | 97988689 | G | A | 0.3041 | -0.122 | 0.0189 | 1.01E-10 | 2.33E-05 | 41.7 |
| rs55747751 | 5 | 132397351 | A | G | 0.081 | -0.2239 | 0.0331 | 1.39E-11 | 8.99E-06 | 45.8 |
| rs55770741 | 5 | 96220087 | T | C | 0.5613 | -0.1281 | 0.0175 | 2.20E-13 | 3.48E-05 | 53.6 |
| rs55857306 | 1 | 11895795 | A | G | 0.1602 | -0.5224 | 0.0235 | 5.05E-109 | 0.000175509 | 494.2 |
| rs55935819 | 12 | 2521579 | A | G | 0.3636 | 0.1271 | 0.0181 | 1.96E-12 | 3.01E-05 | 49.3 |
| rs55944332 | 2 | 145726621 | G | A | 0.2368 | 0.2365 | 0.0204 | 3.27E-31 | 6.41E-05 | 134.4 |
| rs55993676 | 5 | 173303392 | T | G | 0.2916 | -0.2097 | 0.0191 | 3.82E-28 | 6.57E-05 | 120.5 |
| rs56256111 | 13 | 41478963 | A | G | 0.1442 | 0.1926 | 0.0263 | 2.60E-13 | 1.75E-05 | 53.6 |
| rs57708073 | 15 | 79066653 | G | A | 0.2608 | -0.1907 | 0.0214 | 4.73E-19 | 4.04E-05 | 79.4 |
| rs57748895 | 1 | 115826169 | T | A | 0.0179 | 0.6627 | 0.0666 | 2.49E-23 | 4.59E-06 | 99.0 |
| rs58407878 | 7 | 7260161 | A | T | 0.1503 | 0.1569 | 0.0244 | 1.22E-10 | 1.39E-05 | 41.3 |
| rs58693787 | 18 | 48141710 | G | A | 0.2458 | -0.1584 | 0.0202 | 3.82E-15 | 3.01E-05 | 61.5 |
| rs5992929 | 22 | 18451977 | T | C | 0.2834 | 0.1684 | 0.0193 | 3.07E-18 | 4.08E-05 | 76.1 |
| rs602521 | 1 | 227311066 | A | G | 0.2656 | 0.1351 | 0.0195 | 3.97E-12 | 2.47E-05 | 48.0 |
| rs6026739 | 20 | 57739469 | T | A | 0.1226 | 0.5032 | 0.0266 | 1.49E-79 | 0.000101624 | 357.9 |
| rs604723 | 11 | 100610546 | C | T | 0.7247 | 0.3848 | 0.0194 | 2.32E-87 | 0.000207215 | 393.4 |
| rs6058261 | 20 | 30235470 | A | C | 0.2736 | -0.1201 | 0.0195 | 6.83E-10 | 1.99E-05 | 37.9 |
| rs6062295 | 20 | 62291174 | G | A | 0.5744 | 0.1198 | 0.0177 | 1.17E-11 | 2.96E-05 | 45.8 |
| rs6078393 | 20 | 11908101 | G | T | 0.4106 | -0.1205 | 0.0176 | 7.66E-12 | 2.99E-05 | 46.9 |
| rs6108168 | 20 | 8626271 | A | C | 0.2546 | -0.1901 | 0.0199 | 1.10E-21 | 4.57E-05 | 91.3 |
| rs61772592 | 1 | 56979681 | G | A | 0.1257 | 0.1509 | 0.0261 | 7.42E-09 | 9.70E-06 | 33.4 |
| rs61789369 | 4 | 2265295 | G | A | 0.0435 | 0.3039 | 0.0436 | 3.06E-12 | 5.34E-06 | 48.6 |
| rs61909958 | 11 | 96151677 | G | C | 0.1881 | -0.1275 | 0.0228 | 2.21E-08 | 1.26E-05 | 31.3 |
| rs61917655 | 12 | 48210787 | T | C | 0.1011 | 0.2246 | 0.0297 | 3.72E-14 | 1.37E-05 | 57.2 |
| rs61948065 | 13 | 25255052 | C | A | 0.1212 | 0.1737 | 0.027 | 1.17E-10 | 1.16E-05 | 41.4 |
| rs62030049 | 16 | 50572709 | G | A | 0.2404 | -0.1336 | 0.0209 | 1.55E-10 | 1.97E-05 | 40.9 |
| rs62155750 | 2 | 96491456 | G | A | 0.3074 | 0.2177 | 0.0196 | 8.27E-29 | 6.93E-05 | 123.4 |
| rs62158170 | 2 | 114082175 | G | A | 0.2166 | -0.1645 | 0.0211 | 6.63E-15 | 2.72E-05 | 60.8 |
| rs62234672 | 3 | 16592069 | A | C | 0.1752 | 0.1248 | 0.0229 | 4.92E-08 | 1.13E-05 | 29.7 |
| rs62301873 | 4 | 40603821 | G | A | 0.1061 | 0.1734 | 0.0284 | 1.06E-09 | 9.33E-06 | 37.3 |
| rs62378003 | 5 | 89514206 | T | C | 0.1141 | -0.1714 | 0.028 | 8.90E-10 | 1.00E-05 | 37.5 |
| rs62413546 | 6 | 56012664 | T | C | 0.0847 | -0.1877 | 0.032 | 4.58E-09 | 7.04E-06 | 34.4 |
| rs62434124 | 6 | 150999751 | T | C | 0.0711 | -0.4853 | 0.0338 | 7.83E-47 | 3.59E-05 | 206.2 |
| rs62503324 | 8 | 23400615 | T | C | 0.2397 | 0.2033 | 0.0204 | 2.11E-23 | 4.78E-05 | 99.3 |
| rs636202 | 6 | 139843583 | C | T | 0.5185 | -0.1023 | 0.0174 | 4.40E-09 | 2.28E-05 | 34.6 |
| rs6464165 | 7 | 151413124 | C | T | 0.2809 | 0.217 | 0.0195 | 7.34E-29 | 6.60E-05 | 123.8 |
| rs6487076 | 12 | 20470857 | G | A | 0.223 | -0.174 | 0.0209 | 8.69E-17 | 3.17E-05 | 69.3 |
| rs6490019 | 12 | 115920472 | G | A | 0.6203 | 0.1778 | 0.0178 | 2.10E-23 | 6.20E-05 | 99.8 |
| rs6546810 | 2 | 73389716 | C | T | 0.3525 | 0.12 | 0.0181 | 3.16E-11 | 2.65E-05 | 44.0 |
| rs6556384 | 5 | 158418952 | A | C | 0.8105 | -0.152 | 0.0221 | 5.91E-12 | 1.92E-05 | 47.3 |
| rs6580970 | 12 | 54434277 | T | C | 0.2987 | -0.1661 | 0.0191 | 4.03E-18 | 4.18E-05 | 75.6 |
| rs6602177 | 10 | 17167141 | T | C | 0.7073 | -0.1203 | 0.0207 | 6.52E-09 | 1.85E-05 | 33.8 |
| rs66682451 | 11 | 107097540 | G | A | 0.2747 | -0.1348 | 0.0194 | 3.44E-12 | 2.54E-05 | 48.3 |
| rs6686889 | 1 | 25030470 | T | C | 0.2533 | 0.1918 | 0.0199 | 6.95E-22 | 4.64E-05 | 92.9 |
| rs66887589 | 4 | 120509279 | C | T | 0.4779 | 0.161 | 0.0174 | 1.83E-20 | 5.64E-05 | 85.6 |
| rs672272 | 20 | 10488159 | T | C | 0.6041 | -0.1851 | 0.0178 | 2.77E-25 | 6.83E-05 | 108.1 |
| rs675605 | 13 | 110873556 | C | G | 0.7159 | -0.1205 | 0.0196 | 8.46E-10 | 2.03E-05 | 37.8 |
| rs6763931 | 3 | 141102833 | A | G | 0.4438 | 0.1383 | 0.0173 | 1.48E-15 | 4.16E-05 | 63.9 |
| rs6777317 | 3 | 197070959 | A | G | 0.2899 | 0.1249 | 0.0195 | 1.51E-10 | 2.23E-05 | 41.0 |
| rs6779368 | 3 | 185298868 | G | A | 0.3423 | 0.1791 | 0.0184 | 2.28E-22 | 5.63E-05 | 94.7 |
| rs6795735 | 3 | 64705365 | T | C | 0.4109 | -0.1438 | 0.0176 | 3.05E-16 | 4.27E-05 | 66.8 |
| rs6800730 | 3 | 48174210 | G | A | 0.6702 | 0.2476 | 0.0185 | 8.00E-41 | 0.000104521 | 179.1 |
| rs68085857 | 1 | 217737629 | T | C | 0.234 | 0.191 | 0.0205 | 9.82E-21 | 4.11E-05 | 86.8 |
| rs682681 | 13 | 22294062 | C | T | 0.6665 | 0.1454 | 0.0185 | 4.47E-15 | 3.62E-05 | 61.8 |
| rs6875967 | 5 | 50878292 | G | A | 0.6479 | -0.1344 | 0.0181 | 1.21E-13 | 3.32E-05 | 55.1 |
| rs6905288 | 6 | 43758873 | A | G | 0.5681 | 0.1759 | 0.0179 | 7.79E-23 | 6.25E-05 | 96.6 |
| rs6934891 | 6 | 22139729 | A | G | 0.4255 | 0.1275 | 0.0177 | 5.21E-13 | 3.35E-05 | 51.9 |
| rs6961048 | 7 | 27328187 | G | C | 0.1038 | 0.2729 | 0.0286 | 1.28E-21 | 2.24E-05 | 91.0 |
| rs6983239 | 8 | 72507296 | T | G | 0.2188 | 0.1159 | 0.0211 | 3.71E-08 | 1.36E-05 | 30.2 |
| rs7012891 | 8 | 126514676 | C | T | 0.2367 | 0.1391 | 0.0205 | 1.20E-11 | 2.20E-05 | 46.0 |
| rs7106104 | 11 | 111635655 | C | T | 0.281 | 0.1186 | 0.0193 | 7.72E-10 | 2.01E-05 | 37.8 |
| rs710698 | 12 | 70369918 | G | A | 0.4135 | -0.1059 | 0.0176 | 1.88E-09 | 2.32E-05 | 36.2 |
| rs7115331 | 11 | 76218590 | G | T | 0.2857 | 0.1266 | 0.0192 | 3.91E-11 | 2.34E-05 | 43.5 |
| rs7137828 | 12 | 111932800 | T | C | 0.5183 | -0.5027 | 0.0176 | 4.80E-180 | 0.0005377 | 815.8 |
| rs7155504 | 14 | 36158828 | C | T | 0.0876 | -0.2286 | 0.0317 | 5.16E-13 | 1.10E-05 | 52.0 |
| rs7192407 | 16 | 49783926 | C | T | 0.528 | -0.1019 | 0.0174 | 4.53E-09 | 2.26E-05 | 34.3 |
| rs7217916 | 17 | 76769434 | G | A | 0.6146 | -0.1111 | 0.0179 | 5.63E-10 | 2.41E-05 | 38.5 |
| rs722783 | 8 | 120442287 | A | G | 0.2216 | -0.2093 | 0.0208 | 9.03E-24 | 4.61E-05 | 101.3 |
| rs7257694 | 19 | 30314666 | T | C | 0.4003 | 0.1837 | 0.0178 | 6.28E-25 | 6.75E-05 | 106.5 |
| rs7259285 | 19 | 17153149 | A | G | 0.4466 | 0.105 | 0.0176 | 2.65E-09 | 2.32E-05 | 35.6 |
| rs72613227 | 6 | 106320771 | T | A | 0.1269 | 0.1884 | 0.0285 | 3.87E-11 | 1.28E-05 | 43.7 |
| rs7265695 | 20 | 40043096 | C | T | 0.1965 | -0.1967 | 0.0219 | 2.48E-19 | 3.36E-05 | 80.7 |
| rs72683923 | 14 | 50735947 | C | T | 0.0212 | -0.5325 | 0.0635 | 5.02E-17 | 3.85E-06 | 70.3 |
| rs72704264 | 1 | 145713305 | C | G | 0.2172 | 0.117 | 0.0212 | 3.60E-08 | 1.37E-05 | 30.5 |
| rs72719149 | 4 | 144043336 | C | T | 0.3164 | 0.1279 | 0.0186 | 6.34E-12 | 2.70E-05 | 47.3 |
| rs7278003 | 21 | 44966069 | C | T | 0.5615 | 0.1293 | 0.0176 | 1.78E-13 | 3.51E-05 | 54.0 |
| rs72831343 | 10 | 63515681 | G | T | 0.1419 | -0.4936 | 0.0248 | 4.77E-88 | 0.000127338 | 396.1 |
| rs72842207 | 10 | 121433675 | T | C | 0.2149 | -0.2112 | 0.0211 | 1.10E-23 | 4.46E-05 | 100.2 |
| rs72976750 | 4 | 86725684 | C | T | 0.1396 | 0.1718 | 0.0251 | 7.37E-12 | 1.49E-05 | 46.8 |
| rs73036520 | 19 | 45749484 | C | G | 0.2543 | 0.1557 | 0.0202 | 1.34E-14 | 2.97E-05 | 59.4 |
| rs73046792 | 19 | 49605705 | A | G | 0.1592 | -0.1518 | 0.0245 | 5.87E-10 | 1.36E-05 | 38.4 |
| rs7321688 | 13 | 115000365 | A | C | 0.2325 | 0.1507 | 0.0205 | 1.99E-13 | 2.55E-05 | 54.0 |
| rs7324697 | 13 | 58259492 | A | C | 0.325 | 0.1047 | 0.0185 | 1.59E-08 | 1.85E-05 | 32.0 |
| rs73276406 | 8 | 96021760 | C | G | 0.1457 | 0.1564 | 0.0246 | 1.99E-10 | 1.33E-05 | 40.4 |
| rs7350752 | 14 | 21841154 | A | G | 0.1241 | -0.1504 | 0.0268 | 1.97E-08 | 9.04E-06 | 31.5 |
| rs7427249 | 3 | 37572489 | A | G | 0.58 | -0.1098 | 0.0176 | 4.34E-10 | 2.50E-05 | 38.9 |
| rs74439044 | 17 | 7781019 | C | T | 0.0983 | 0.3496 | 0.0294 | 1.38E-32 | 3.31E-05 | 141.4 |
| rs7491960 | 13 | 114470370 | T | C | 0.492 | -0.1288 | 0.018 | 8.44E-13 | 3.38E-05 | 51.2 |
| rs751984 | 11 | 61278246 | C | T | 0.1174 | -0.3937 | 0.0275 | 1.38E-46 | 5.61E-05 | 205.0 |
| rs7524019 | 1 | 167367193 | T | C | 0.492 | 0.1036 | 0.0174 | 2.60E-09 | 2.34E-05 | 35.5 |
| rs75511781 | 7 | 131323710 | G | A | 0.0425 | 0.3721 | 0.047 | 2.45E-15 | 6.73E-06 | 62.7 |
| rs7569128 | 2 | 191446691 | A | C | 0.8185 | 0.1981 | 0.0225 | 1.22E-18 | 3.04E-05 | 77.5 |
| rs75717699 | 2 | 179682997 | G | T | 0.0305 | 0.4667 | 0.0541 | 6.71E-18 | 5.81E-06 | 74.4 |
| rs7572130 | 2 | 164460947 | G | A | 0.1042 | 0.1796 | 0.0287 | 4.12E-10 | 9.65E-06 | 39.2 |
| rs7611674 | 3 | 179169230 | G | T | 0.1962 | -0.1576 | 0.0223 | 1.67E-12 | 2.08E-05 | 49.9 |
| rs7623706 | 3 | 74712754 | G | A | 0.4349 | -0.0975 | 0.0176 | 2.83E-08 | 1.99E-05 | 30.7 |
| rs76326501 | 2 | 43167878 | C | A | 0.0911 | -0.3618 | 0.0305 | 2.17E-32 | 3.08E-05 | 140.7 |
| rs76452347 | 9 | 35906471 | T | C | 0.2053 | -0.2246 | 0.0229 | 9.37E-23 | 4.14E-05 | 96.2 |
| rs76719272 | 1 | 156129796 | T | C | 0.1315 | -0.1438 | 0.0264 | 4.86E-08 | 8.95E-06 | 29.7 |
| rs76785130 | 6 | 121813835 | G | A | 0.0199 | 0.4285 | 0.0662 | 9.36E-11 | 2.16E-06 | 41.9 |
| rs76954792 | 17 | 30033514 | T | C | 0.2322 | 0.1213 | 0.0208 | 5.06E-09 | 1.60E-05 | 34.0 |
| rs77032376 | 15 | 90010780 | T | C | 0.1479 | -0.173 | 0.0249 | 3.64E-12 | 1.61E-05 | 48.3 |
| rs7737851 | 5 | 42415845 | C | T | 0.8056 | 0.1256 | 0.022 | 1.11E-08 | 1.35E-05 | 32.6 |
| rs7788746 | 7 | 99612405 | T | G | 0.6691 | -0.1644 | 0.0183 | 3.19E-19 | 4.72E-05 | 80.7 |
| rs77924615 | 16 | 20392332 | A | G | 0.1982 | -0.3163 | 0.0224 | 3.72E-45 | 8.36E-05 | 199.4 |
| rs7800558 | 7 | 140242661 | C | T | 0.4219 | -0.096 | 0.0175 | 4.46E-08 | 1.94E-05 | 30.1 |
| rs78151625 | 3 | 158316726 | C | T | 0.1658 | 0.1869 | 0.0233 | 1.04E-15 | 2.35E-05 | 64.3 |
| rs786921 | 1 | 89286673 | A | G | 0.5957 | -0.1145 | 0.0176 | 8.63E-11 | 2.69E-05 | 42.3 |
| rs78809139 | 3 | 154674943 | A | G | 0.1014 | -0.2281 | 0.0288 | 2.58E-15 | 1.51E-05 | 62.7 |
| rs78909293 | 5 | 148335250 | C | T | 0.0449 | -0.321 | 0.0429 | 7.31E-14 | 6.34E-06 | 56.0 |
| rs79044887 | 20 | 47427831 | G | C | 0.1476 | -0.2427 | 0.0245 | 4.01E-23 | 3.26E-05 | 98.1 |
| rs7926335 | 11 | 16917869 | T | C | 0.2699 | 0.1804 | 0.0195 | 2.05E-20 | 4.45E-05 | 85.6 |
| rs79286081 | 16 | 86555837 | A | G | 0.1021 | -0.1631 | 0.0299 | 4.83E-08 | 7.20E-06 | 29.8 |
| rs7933758 | 11 | 31000774 | T | C | 0.3047 | -0.1138 | 0.0191 | 2.58E-09 | 1.99E-05 | 35.5 |
| rs7938342 | 11 | 1887806 | A | T | 0.5877 | 0.2672 | 0.0181 | 1.92E-49 | 0.000139404 | 217.9 |
| rs7967705 | 12 | 50511408 | C | T | 0.6196 | -0.2694 | 0.0178 | 1.54E-51 | 0.000142527 | 229.1 |
| rs79724577 | 17 | 43463493 | C | A | 0.1832 | -0.1362 | 0.023 | 3.47E-09 | 1.39E-05 | 35.1 |
| rs79889784 | 11 | 1702117 | T | G | 0.0176 | -0.3941 | 0.0717 | 3.86E-08 | 1.38E-06 | 30.2 |
| rs7990017 | 13 | 110959705 | T | C | 0.4733 | 0.1039 | 0.0185 | 1.92E-08 | 2.08E-05 | 31.5 |
| rs7992292 | 13 | 41968013 | A | G | 0.824 | 0.1367 | 0.0231 | 3.19E-09 | 1.34E-05 | 35.0 |
| rs80095680 | 16 | 30902353 | G | A | 0.2633 | 0.1566 | 0.0198 | 2.81E-15 | 3.20E-05 | 62.6 |
| rs8014182 | 14 | 103859962 | T | C | 0.1319 | -0.1942 | 0.0257 | 3.94E-14 | 1.73E-05 | 57.1 |
| rs8081301 | 17 | 47039302 | T | A | 0.2705 | -0.1278 | 0.0197 | 8.67E-11 | 2.19E-05 | 42.1 |
| rs8108717 | 19 | 31911914 | G | A | 0.6084 | -0.1323 | 0.0179 | 1.39E-13 | 3.44E-05 | 54.6 |
| rs824523 | 2 | 19707855 | A | C | 0.3344 | 0.1226 | 0.0183 | 2.26E-11 | 2.64E-05 | 44.9 |
| rs881858 | 6 | 43806609 | A | G | 0.694 | 0.1553 | 0.0191 | 4.65E-16 | 3.71E-05 | 66.1 |
| rs882624 | 1 | 201735913 | T | C | 0.3325 | -0.1571 | 0.0185 | 2.33E-17 | 4.23E-05 | 72.1 |
| rs908951 | 16 | 89697625 | T | C | 0.437 | -0.1983 | 0.0181 | 7.73E-28 | 7.80E-05 | 120.0 |
| rs917522 | 16 | 4097222 | T | C | 0.885 | 0.1665 | 0.0273 | 1.04E-09 | 9.99E-06 | 37.2 |
| rs926335 | 22 | 28046423 | T | C | 0.4764 | -0.1215 | 0.0175 | 3.37E-12 | 3.17E-05 | 48.2 |
| rs9286351 | 4 | 138441530 | G | A | 0.4188 | 0.1412 | 0.0177 | 1.61E-15 | 4.09E-05 | 63.6 |
| rs9289557 | 3 | 138071604 | T | C | 0.2604 | -0.119 | 0.0207 | 8.68E-09 | 1.68E-05 | 33.0 |
| rs9326869 | 5 | 112349070 | C | T | 0.7513 | -0.1096 | 0.02 | 3.99E-08 | 1.48E-05 | 30.0 |
| rs9365555 | 6 | 163757127 | G | A | 0.3259 | -0.1254 | 0.0187 | 1.96E-11 | 2.61E-05 | 45.0 |
| rs9399137 | 6 | 135419018 | C | T | 0.2619 | -0.1148 | 0.0197 | 5.83E-09 | 1.73E-05 | 34.0 |
| rs9406076 | 6 | 8023804 | T | C | 0.3278 | 0.101 | 0.0185 | 4.65E-08 | 1.73E-05 | 29.8 |
| rs9419374 | 10 | 133729749 | G | A | 0.646 | -0.1164 | 0.0185 | 3.44E-10 | 2.39E-05 | 39.6 |
| rs9467545 | 6 | 25638464 | T | A | 0.1572 | 0.2545 | 0.0237 | 7.39E-27 | 4.03E-05 | 115.3 |
| rs9478282 | 6 | 152398669 | T | C | 0.1116 | -0.1994 | 0.0279 | 8.70E-13 | 1.34E-05 | 51.1 |
| rs9508495 | 13 | 30146201 | T | C | 0.7569 | -0.1944 | 0.0204 | 1.34E-21 | 4.41E-05 | 90.8 |
| rs951914 | 8 | 25878995 | C | G | 0.7126 | 0.1904 | 0.0193 | 5.06E-23 | 5.26E-05 | 97.3 |
| rs9526707 | 13 | 51489186 | A | G | 0.3222 | -0.1217 | 0.0186 | 6.59E-11 | 2.47E-05 | 42.8 |
| rs962369 | 11 | 27734420 | C | T | 0.3013 | -0.1684 | 0.0189 | 6.02E-19 | 4.41E-05 | 79.4 |
| rs9638084 | 7 | 156311745 | G | A | 0.6022 | -0.1154 | 0.0178 | 8.51E-11 | 2.66E-05 | 42.0 |
| rs964941 | 1 | 228193857 | A | G | 0.5183 | 0.1726 | 0.0174 | 3.31E-23 | 6.49E-05 | 98.4 |
| rs9791312 | 6 | 143142313 | C | A | 0.3452 | 0.1225 | 0.0184 | 2.89E-11 | 2.64E-05 | 44.3 |
| rs9841978 | 3 | 53730735 | A | G | 0.3251 | 0.1766 | 0.0185 | 1.12E-21 | 5.28E-05 | 91.1 |
| rs9889262 | 17 | 47398070 | A | T | 0.3666 | 0.2283 | 0.018 | 7.11E-37 | 9.86E-05 | 160.9 |
| rs9918907 | 8 | 124816862 | G | A | 0.2162 | 0.1188 | 0.021 | 1.59E-08 | 1.43E-05 | 32.0 |
| rs9932220 | 16 | 51758116 | A | G | 0.2177 | -0.1591 | 0.021 | 3.76E-14 | 2.58E-05 | 57.4 |
| rs9937801 | 16 | 21088130 | C | T | 0.4308 | -0.1554 | 0.0174 | 4.81E-19 | 5.16E-05 | 79.8 |

**SNP: single nucleotide polymorphism; CHR: chromosome; EAF: effect allele frequency; SE: standard error.**
