# Supplementary material for: Hypertension, antihypertensive drugs, and age at onset of Huntington’s disease
Source: Orphanet J Rare Dis. 2023 May 24;18:125. doi: 10.1186/s13023-023-02734-1 (PMC10207760; doi:10.1186/s13023-023-02734-1)
Supplement: Supplementary file 3 — Supplementary Material 3 [file 13023_2023_2734_MOESM3_ESM.docx]

**Table S3 Single nucleotide polymorphisms (SNP) that fulfilled our selection criteria to be used as proxies for the effects for antihypertensive drug classes**

| **SNP** | **Chr** | **Position** | **Gene** | **Effect allele** | **EAF** | **Beta** | **SE** | **P-value** | **R^2^** | **F** |
| --- | --- | --- | --- | --- | --- | --- | --- | --- | --- | --- |
| **ACE inhibitors** | | | | | | | | | | |
| rs4291 | 17 | 61554194 | ACE | A | 0.615 | -0.2839 | 0.0312 | 8.65E-20 | 0.00037 | 82.8 |
| **Beta blockers** | | | | | | | | | | |
| rs11196549^a^ | 10 | 115707298 | ADRB1 | A | 0.042 | 0.6884 | 0.0784 | 1.58E-18 | 0.00015 | 77.1 |
| rs460718^a^ | 10 | 115721364 | ADRB1 | A | 0.326 | -0.2764 | 0.0324 | 1.36E-17 | 0.00033 | 72.8 |
| rs11196597^a^ | 10 | 115788094 | ADRB1 | A | 0.133 | 0.2858 | 0.0458 | 4.23E-10 | 0.00018 | 38.9 |
| rs79850079^a^ | 10 | 115790006 | ADRB1 | A | 0.031 | -0.5804 | 0.0905 | 1.45E-10 | 9.80E-05 | 41.1 |
| rs17875473^a^ | 10 | 115800294 | ADRB1 | T | 0.087 | 0.3283 | 0.0552 | 2.66E-09 | 0.00014 | 35.4 |
| rs2429511 | 10 | 115801253 | ADRB1 | T | 0.52 | -0.3728 | 0.0303 | 7.39E-35 | 0.00051 | 151.4 |
| rs1801253^a^ | 10 | 115805056 | ADRB1 | C | 0.733 | 0.4626 | 0.0344 | 2.84E-41 | 5.00E-04 | 180.8 |
| rs4359161^a^ | 10 | 115826508 | ADRB1 | A | 0.181 | -0.2662 | 0.0391 | 9.46E-12 | 0.00022 | 46.4 |
| **Calcium channel blockers** | | | | | | | | | | |
| rs116556102 | 12 | 2303850 | CACNA1C | C | 0.983 | -0.6853 | 0.1252 | 4.42E-08 | 6.00E-05 | 30.0 |
| rs2239046^a^ | 12 | 2434419 | CACNA1C | A | 0.681 | 0.2082 | 0.0322 | 9.58E-11 | 0.00025 | 41.8 |
| rs714277^a^ | 12 | 2514270 | CACNA1C | T | 0.283 | 0.1986 | 0.0333 | 2.38E-09 | 0.00022 | 35.6 |
| rs2488136^a^ | 10 | 18334521 | CACNB2 | A | 0.287 | 0.2261 | 0.0334 | 1.22E-11 | 0.00025 | 45.8 |
| rs1888693^a^ | 10 | 18440444 | CACNB2 | A | 0.344 | 0.3858 | 0.0317 | 4.69E-34 | 0.00048 | 148.1 |
| rs17604757^a^ | 10 | 18442940 | CACNB2 | A | 0.932 | -0.5022 | 0.0606 | 1.12E-16 | 0.00017 | 68.7 |
| rs12571593 | 10 | 18443222 | CACNB2 | A | 0.907 | -0.3996 | 0.0521 | 1.71E-14 | 0.00018 | 58.8 |
| rs12414844 | 10 | 18451994 | CACNB2 | T | 0.266 | 0.279 | 0.0342 | 3.15E-16 | 3.00E-04 | 66.6 |
| rs7076319^a^ | 10 | 18459450 | CACNB2 | A | 0.733 | -0.321 | 0.0341 | 5.07E-21 | 0.00034 | 88.6 |
| rs17662793^a^ | 10 | 18465479 | CACNB2 | A | 0.712 | 0.2363 | 0.0338 | 2.65E-12 | 0.00027 | 48.9 |
| rs10828295 | 10 | 18466094 | CACNB2 | A | 0.747 | -0.3397 | 0.0349 | 2.18E-22 | 0.00035 | 94.7 |
| rs16916922 | 10 | 18467744 | CACNB2 | A | 0.858 | -0.3662 | 0.0433 | 2.86E-17 | 0.00024 | 71.5 |
| rs61278674^a^ | 10 | 18481737 | CACNB2 | A | 0.906 | -0.3298 | 0.054 | 1.03E-09 | 0.00015 | 37.3 |
| rs4748444 | 10 | 18494482 | CACNB2 | T | 0.663 | 0.1939 | 0.0327 | 3.13E-09 | 0.00024 | 35.2 |
| rs1539680^a^ | 10 | 18502889 | CACNB2 | C | 0.792 | -0.3259 | 0.0375 | 3.37E-18 | 0.00029 | 75.5 |
| rs1779209 | 10 | 18514561 | CACNB2 | T | 0.287 | 0.2736 | 0.0336 | 4.23E-16 | 0.00031 | 66.3 |
| rs1757213^a^ | 10 | 18537594 | CACNB2 | A | 0.112 | 0.3084 | 0.0507 | 1.15E-09 | 0.00017 | 37.0 |
| rs10828399^a^ | 10 | 18553968 | CACNB2 | A | 0.521 | -0.1947 | 0.0302 | 1.10E-10 | 0.00027 | 41.6 |
| rs10828452^a^ | 10 | 18592450 | CACNB2 | A | 0.793 | 0.3046 | 0.0388 | 4.20E-15 | 0.00027 | 61.6 |
| rs17610275 | 10 | 18621630 | CACNB2 | T | 0.926 | 0.3868 | 0.0613 | 2.87E-10 | 0.00014 | 39.8 |
| rs10828542^a^ | 10 | 18627285 | CACNB2 | A | 0.613 | 0.1817 | 0.0311 | 5.18E-09 | 0.00024 | 34.1 |
| rs112701401^a^ | 10 | 18644811 | CACNB2 | C | 0.969 | 0.5026 | 0.0921 | 4.92E-08 | 8.20E-05 | 29.8 |
| rs7072277 | 10 | 18658707 | CACNB2 | A | 0.471 | -0.1746 | 0.0301 | 6.88E-09 | 0.00024 | 33.6 |
| rs11013938 | 10 | 18669271 | CACNB2 | C | 0.255 | -0.3265 | 0.035 | 1.17E-20 | 0.00034 | 87.0 |
| rs12780039 | 10 | 18678987 | CACNB2 | C | 0.121 | 0.2852 | 0.047 | 1.26E-09 | 0.00017 | 36.8 |
| rs79253631 | 10 | 18694223 | CACNB2 | A | 0.986 | -0.7774 | 0.1392 | 2.32E-08 | 5.70E-05 | 31.2 |
| rs112133583 | 10 | 18695681 | CACNB2 | T | 0.029 | -0.5546 | 0.0973 | 1.18E-08 | 8.80E-05 | 32.5 |
| rs7909027^a^ | 10 | 18695892 | CACNB2 | T | 0.649 | -0.3312 | 0.0318 | 2.01E-25 | 0.00041 | 108.5 |
| rs10828662 | 10 | 18703097 | CACNB2 | T | 0.558 | -0.2879 | 0.0304 | 2.54E-21 | 0.00039 | 89.7 |
| rs982003^a^ | 10 | 18707296 | CACNB2 | T | 0.756 | -0.2414 | 0.0351 | 6.21E-12 | 0.00024 | 47.3 |
| rs1325990 | 10 | 18707352 | CACNB2 | A | 0.47 | -0.3873 | 0.0302 | 1.09E-37 | 0.00053 | 164.5 |
| rs11014170 | 10 | 18710991 | CACNB2 | A | 0.02 | -0.6701 | 0.115 | 5.61E-09 | 7.40E-05 | 34.0 |
| rs72786085^a^ | 10 | 18713206 | CACNB2 | C | 0.079 | -0.5309 | 0.0595 | 4.46E-19 | 0.00021 | 79.6 |
| rs10828689 | 10 | 18721957 | CACNB2 | C | 0.443 | -0.3634 | 0.0304 | 6.94E-33 | 0.00049 | 142.9 |
| rs67214975 | 10 | 18727251 | CACNB2 | A | 0.456 | -0.4144 | 0.0307 | 1.42E-41 | 0.00057 | 182.2 |
| rs7923191 | 10 | 18727901 | CACNB2 | A | 0.791 | -0.369 | 0.0376 | 1.10E-22 | 0.00033 | 96.3 |
| rs12258967^a^ | 10 | 18727959 | CACNB2 | C | 0.704 | 0.6327 | 0.0337 | 1.08E-78 | 0.00072 | 352.5 |
| rs72786098 | 10 | 18729855 | CACNB2 | A | 0.032 | -0.5033 | 0.0883 | 1.18E-08 | 8.60E-05 | 32.5 |
| rs116936375^a^ | 10 | 18737135 | CACNB2 | A | 0.04 | -0.5739 | 0.081 | 1.40E-12 | 0.00012 | 50.2 |
| rs12256244 | 10 | 18750045 | CACNB2 | A | 0.626 | 0.4246 | 0.0315 | 2.09E-41 | 0.00055 | 181.7 |
| rs1998822^a^ | 10 | 18755664 | CACNB2 | A | 0.723 | -0.1958 | 0.0343 | 1.15E-08 | 0.00022 | 32.6 |
| rs7070582 | 10 | 18755942 | CACNB2 | T | 0.383 | 0.2502 | 0.0317 | 2.86E-15 | 0.00033 | 62.3 |
| rs7076100 | 10 | 18759537 | CACNB2 | A | 0.406 | -0.3569 | 0.0308 | 5.51E-31 | 0.00047 | 134.3 |
| rs7076247^a^ | 10 | 18759629 | CACNB2 | T | 0.388 | 0.2557 | 0.0309 | 1.33E-16 | 0.00033 | 68.5 |
| rs11014494 | 10 | 18780705 | CACNB2 | A | 0.492 | 0.1676 | 0.0304 | 3.37E-08 | 0.00023 | 30.4 |
| rs10828784^a^ | 10 | 18788273 | CACNB2 | C | 0.663 | 0.2021 | 0.0345 | 4.49E-09 | 0.00025 | 34.3 |
| rs12416030^a^ | 10 | 18789075 | CACNB2 | T | 0.796 | -0.2088 | 0.0381 | 4.32E-08 | 0.00019 | 30.0 |
| rs12416052^a^ | 10 | 18789267 | CACNB2 | T | 0.594 | 0.1987 | 0.0311 | 1.59E-10 | 0.00026 | 40.8 |
| rs4748476 | 10 | 18792875 | CACNB2 | T | 0.777 | 0.2166 | 0.0365 | 2.89E-09 | 0.00021 | 35.2 |
| rs150857355^a^ | 12 | 49209340 | CACNB3 | C | 0.021 | 0.9406 | 0.1122 | 5.20E-17 | 0.00011 | 70.3 |
| rs312487 | 3 | 53545622 | CACNA1D | T | 0.478 | 0.2194 | 0.0307 | 9.65E-13 | 3.00E-04 | 51.1 |
| rs3821843^a^ | 3 | 53558012 | CACNA1D | A | 0.68 | 0.3373 | 0.0335 | 6.56E-24 | 4.00E-04 | 101.4 |
| rs9311502^a^ | 3 | 53560321 | CACNA1D | T | 0.76 | -0.2463 | 0.0355 | 3.87E-12 | 0.00025 | 48.1 |
| rs1547950 | 3 | 53568283 | CACNA1D | T | 0.537 | -0.2151 | 0.0307 | 2.33E-12 | 0.00029 | 49.1 |
| rs11709630 | 3 | 53577164 | CACNA1D | T | 0.637 | 0.1931 | 0.032 | 1.61E-09 | 0.00025 | 36.4 |
| rs114987861^a^ | 3 | 53605712 | CACNA1D | A | 0.028 | 0.5289 | 0.0958 | 3.36E-08 | 8.00E-05 | 30.5 |
| rs113210396^a^ | 3 | 53612327 | CACNA1D | T | 0.045 | -0.4338 | 0.077 | 1.76E-08 | 1.00E-04 | 31.7 |
| rs3774475 | 3 | 53650483 | CACNA1D | A | 0.417 | 0.1854 | 0.0307 | 1.60E-09 | 0.00025 | 36.5 |
| rs7340705^a^ | 3 | 53734443 | CACNA1D | T | 0.673 | -0.2425 | 0.0322 | 4.87E-14 | 0.00029 | 56.7 |
| rs2633731 | 3 | 53738424 | CACNA1D | T | 0.396 | -0.1963 | 0.0309 | 2.21E-10 | 0.00026 | 40.4 |

**^a^ SNPs also included in the additional analysis of the instruments correlated at a lower LD threshold (r^2^<0.2).**

**ACE: angiotensin converting enzyme; CHR: chromosome; EAF: effect allele frequency; SE: standard error.**
